# Whole-genome and genome-wide association studies improve key agricultural traits of safflower for industrial and medicinal use

**DOI:** 10.1093/hr/uhad197

**Published:** 2023-09-29

**Authors:** Jiang Chen, Shuai Guo, Xueli Hu, Rui Wang, Donghai Jia, Qiang Li, Xianmei Yin, Xuejiao Liao, Zunhong Hu, Peiqi Wang, Chaoxiang Ren, Shuai Dong, Chao Chen, Shilin Chen, Jiang Xu, Jin Pei

**Affiliations:** State Key Laboratory of Southwestern Chinese Medicine Resources, College of Pharmacy, Chengdu University of Traditional Chinese Medicine, Chengdu 611137, China; State Key Laboratory of Southwestern Chinese Medicine Resources, College of Pharmacy, Chengdu University of Traditional Chinese Medicine, Chengdu 611137, China; Institute of Chinese Materia Medica, China Academy of Chinese Medical Sciences, Beijing 100700, China; Industrial Crops Research Institute, Yunnan Academy of Agricultural Sciences, Kunming 650205, China; State Key Laboratory of Southwestern Chinese Medicine Resources, College of Pharmacy, Chengdu University of Traditional Chinese Medicine, Chengdu 611137, China; Institute of Economic Crops, Xinjiang Academy of Agricultural Sciences, Urumchi 830091, China; Institute of Economic Crops, Xinjiang Academy of Agricultural Sciences, Urumchi 830091, China; State Key Laboratory of Southwestern Chinese Medicine Resources, College of Pharmacy, Chengdu University of Traditional Chinese Medicine, Chengdu 611137, China; State Key Laboratory of Southwestern Chinese Medicine Resources, College of Pharmacy, Chengdu University of Traditional Chinese Medicine, Chengdu 611137, China; Institute of Chinese Materia Medica, China Academy of Chinese Medical Sciences, Beijing 100700, China; Industrial Crops Research Institute, Yunnan Academy of Agricultural Sciences, Kunming 650205, China; Industrial Crops Research Institute, Yunnan Academy of Agricultural Sciences, Kunming 650205, China; State Key Laboratory of Southwestern Chinese Medicine Resources, College of Pharmacy, Chengdu University of Traditional Chinese Medicine, Chengdu 611137, China; State Key Laboratory of Southwestern Chinese Medicine Resources, College of Pharmacy, Chengdu University of Traditional Chinese Medicine, Chengdu 611137, China; State Key Laboratory of Southwestern Chinese Medicine Resources, College of Pharmacy, Chengdu University of Traditional Chinese Medicine, Chengdu 611137, China; State Key Laboratory of Southwestern Chinese Medicine Resources, College of Pharmacy, Chengdu University of Traditional Chinese Medicine, Chengdu 611137, China; Institute of Chinese Materia Medica, China Academy of Chinese Medical Sciences, Beijing 100700, China; Institute of Chinese Materia Medica, China Academy of Chinese Medical Sciences, Beijing 100700, China; State Key Laboratory of Southwestern Chinese Medicine Resources, College of Pharmacy, Chengdu University of Traditional Chinese Medicine, Chengdu 611137, China

## Abstract

Safflower (*Carthamus tinctorius*) is widely cultivated around the world for its seeds and flowers. The presence of linoleic acid (LA) in its seeds and hydroxysafflor yellow A (HSYA) in its flowers are the crucial traits that enable safflower to be used for industrial and medicinal purposes. Understanding the genetic control of these traits is essential for optimizing the quality of safflower and its breeding. To further this research, we present a chromosome-scale assembly of the genome of the safflower variety ‘Chuanhonghua 1’, which was achieved using an integrated strategy combining Illumina, Oxford Nanopore, and Hi-C sequencing. We obtained a 1.17-Gb assembly with a contig N50 of 1.08 Mb, and all assembled sequences were assigned to 12 pseudochromosomes. Safflower’s evolution involved the core eudicot γ-triplication event and a whole-genome duplication event, which led to large-scale genomic rearrangements. Extensive genomic shuffling has occurred since the divergence of the ancestor of dicotyledons. We conducted metabolite and transcriptome profiles with time- and part-dependent changes and screened candidate genes that significantly contribute to seed lipid biosynthesis. We also analyzed key gene families that participate in LA and HSYA biosynthesis. Additionally, we re-sequenced 220 safflower lines and carried out a genome-wide association study using high-quality SNP data for eight agronomic traits. We identified SNPs related to important traits in safflower. Besides, the candidate gene *HH_034464* (*CtCGT1*) was shown to be involved in the biosynthesis of HSYA. Overall, we provide a high-quality reference genome and elucidate the genetic basis of LA and HSYA biosynthesis in safflower. This vast amount of data will benefit further research for functional gene mining and breeding in safflower.

## Introduction

Safflower (*Carthamus tinctorius*), belonging to the Compositae or Asteraceae family, is a diploid plant that has been cultivated for ~4000 years in the fertile crescent region [1]. This annual plant is self-compatible and is extensively grown worldwide for its seeds and flowers, which serve various purposes in different fields. Safflower seed oil, rich in linoleic acid (LA), is highly regarded as a premium edible oil due to its beneficial properties [2, 3]. The flowers of safflower are widely utilized across the globe in dyes, cosmetics, and food additives [4, 5]. Furthermore, dried safflower flowers have been traditionally employed in Chinese and Southeast Asian traditional medicine for treating diverse ailments [6, 7]. Safflower contains a bioactive compound called hydroxysafflor yellow A (HSYA) [8, 9, 65]. With its significant potential, safflower can become a noteworthy economic crop [10, 11].

**Figure 1:**
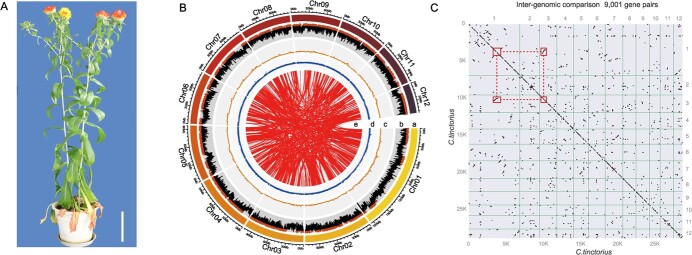
Safflower genome assembly. **A** Photograph of safflower ‘Chuanhonghua 1’. Bar = 10 cm. **B** Concentric circles in the diagram represent different aspects of the genome. From the outermost to the innermost layer, they represent chromosome length (a), repeat content (b), gene density (c), GC content (d, %), and syntenic blocks (e). Regions with repeat content surpassing the third quartile are depicted in brown, while those with above-average GC content are indicated in blue. (C) Dot plot of syntenic blocks in safflower genome.

Safflower is widely cultivated in Asia, Europe, Australia, and the Americas as a versatile crop (http://www.fao.org/faostat/en/#home). As a drought-resistant crop, safflower is expected to play a more important role as global warming and local drought conditions persist. In the different regions where safflower is cultivated, distinct varieties with unique traits have emerged over time. However, the genetic analysis of safflower has been limited, with only a few types of molecular marker being used to assess genetic diversity [12–14]. A safflower variety (‘Anhui-1’) has been sequenced *de novo* using second-generation and third-generation sequencing [15], providing an invaluable genomic resource for genetic diversity and analysis. However, a single reference genome cannot capture all the genes of the species [16–18]. ‘Chuanhonghua 1’ is an ancient variety cultivated in Sichuan, China, with unique agronomic traits, such as its shorter growth period (Fig. 1A). *De novo* sequencing and deep analysis are necessary to explore the genetic resources it offers.

Safflower is highly regarded for its oil, which contains LA, and its flower, which contains flavonoids, particularly HSYA. Both these components have various industrial and medicinal uses. The biochemical process of oil biosynthesis in the form of triacylglycerols (TAGs) is well established in oilseed plants. It involves key enzymes such as diacylglycerol acyltransferase (DGAT) and fatty acid desaturases (FADs). DGAT plays a crucial role in TAG biosynthesis, while FADs convert oleic acid (OA) into LA [19–22]. However, the precise genes responsible for LA biosynthesis in safflower remain incompletely understood. Similarly, although the biosynthetic pathway for flavonoids has been widely documented [23, 24], the specific process for HSYA, the bioactive compound, is still unclear. Recent research suggests a possible involvement of C-glucuronosyltransferases (CGT) and P450 enzymes in HSYA biosynthesis [25]. Hence, comprehensive genome-wide screening is needed to identify potential candidate genes associated with LA and HSYA production.

**Table 1:** Statistics of the safflower genome.

	**Number**	**Size**
Contig assembly features		
Sequence number	3941	
Total bases (bp)		1 170 951 068
Minimum sequence length (bp)		4024
Maximum sequence length (bp)		9 019 709
Average sequence length (bp)		297 120.29
N50 (bp)		1 078 450
(G + C)s (%)		38.41
Chromosome assembly features		
Sequence number	543	
Total bases (bp)		1 174 349 068
Minimum sequence length (bp)		4024
Maximum sequence length (bp)		185 004 703
Average sequence length (bp)		18 099 009.73
N50 (bp)		96 393 662
(G + C)s (%)		38.30
Ns (%)		0.29
Genome annotation		
Gene number	39 809	
Total gene length (bp)		213 400 340
Average gene length (bp)		5360.61
Exon number	235 816	
Total exon length (bp)		55 574 446
Average exon length (bp)		235.669

We employed an integrated methodology to achieve a chromosome-level genome assembly of an ancient native safflower strain, ‘Chuanhonghua 1’. In addition, we conducted metabolite analyses of the seed and flower, as well as transcriptome profiles that reflect time- and organ-specific variations, and combined these analyses to identify key genes that significantly contribute to lipid biosynthesis. We conducted a genomic survey of critical gene families involved in LA and HSYA biosynthesis in seeds and flowers, respectively. We re-sequenced 220 safflower strains and conducted genome-wide association studies (GWAS) using high-quality SNPs to investigate eight crucial agricultural traits. Besides, we designed experiments to demonstrate the function of the screened candidate genes. This extensive data will be invaluable for future research into functional gene mining and safflower breeding for industrial and medicinal purposes.

## Results

### Genomic characteristics of safflower

We sequenced the genome of ‘Chuanhonghua 1’ safflower using an integrated approach that combined Illumina, Oxford Nanopore, and Hi-C sequencing (depicted in Supplementary Data Fig. S1). The Nanopore GridION platform generated roughly 111.45 Gb subreads, providing coverage ~100 times the safflower’s estimated genome size (roughly 1.17 Gb, based on Illumina *k*-mer analysis) (as shown in Supplementary Data Fig. S2 and Supplementary Data Table S1). Filtered reads were initially assembled and refined, resulting in a 1.17-Gb assembly with a contig N50 of 1.08 Mb and a GC content of 38.41% (detailed in Table 1). After Hi-C ligation, the assembled sequences were assigned to 12 chromosomes (50.84–185.00 Mb) with an N50 of 96.39 Mb, consistent with cytogenetic karyotyping methods (Fig. 1B and Supplementary Data Fig. S3). The safflower genome’s average GC content was around 38.30%, showing minimal variation among different chromosomes, with chromosome 10 having the highest GC content (39.08%) and chromosome 1 displaying the lowest (36.89%) (Supplementary Data Table S2). Using the BUSCO method, we verified the assembly’s quality, with 92.36% successful mapping, 97.2% complete genes (89.79% of total), and 91.42% single-copy mapping (84.44% of total) (Supplementary Data Table S3), demonstrating the assembly’s integrity, continuity, and precision. We observed numerous duplicated synteny blocks in the safflower genome (Fig. 1C), leading us to hypothesize that safflower underwent a recent whole-genome duplication (WGD) event.

**Figure 2:**
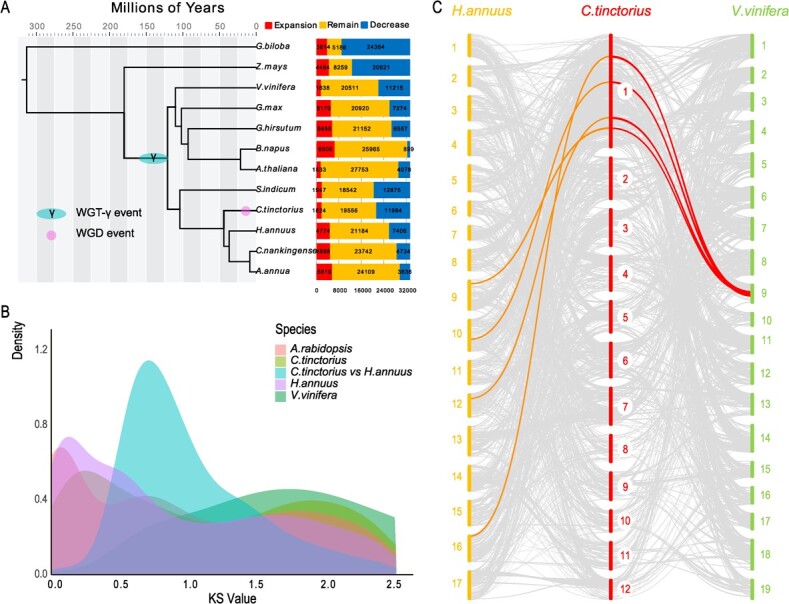
Evolutionary history of safflower. **A** The phylogenetic tree was constructed using 274 single-copy genes from 12 species. All branches in the tree had posterior probabilities exceeding 0.99. The study estimated the timing of safflower WGDs, and other reported whole-genome triplication (WGT)/WGD events are labeled on the tree. Bar charts represent the expansion and reduction of gene families. **B** Distribution of synonymous substitution rates (*K*_s_) for paired syntenic paralogs was analyzed in safflower and three other plant species. **C** Synteny maps were generated to compare safflower, *H. annuus*, and *V. vinifera*. Light gray lines represent synteny blocks. Colors of connecting lines indicate different WGD events among the species. Red lines indicate more duplication events in safflower compared with *V. vinifera*. Orange lines indicate simultaneous WGD events in safflower and *H. annuus*.

We utilized a combination pipeline of *ab initio* and comparison methods to predict a total of 39 809 protein-coding genes. Their average length was ~5.36 kb, while their average coding region was ~1.39 kb (Table 1). Over 70% of these predicted genes were annotated with public databases, such as GO, KO, NR, and Pfam, among others (Supplementary Data Table S4). To elaborate, 23 251 genes (58.16%) were annotated with the GO database, 3613 genes (9.08%) with the KO database, 14 774 genes (37.11%) with the NR database, and 23 246 genes (58.39%) with the PFAM database. Repetitive sequences constituted ~836.16 Mb (71.41%) of the 1.17-Gb sequence. Interestingly, 39.81% (466.20 Mb) of these sequences were identified as long terminal repeat (LTR) elements. LTR elements comprise two primary subfamilies, namely Ty1/Copia (215.99 Mb, 46.33%) and Gypsy/DIRS1 (245.91 Mb, 52.75%) (Supplementary Data Table S5).

### Genome evolution of safflower

We compared the predicted safflower genome with those of 11 other sequenced plants and clustered a total of 33 366 gene families, including 891 unique gene families for safflower specifically (Supplementary Data Fig. S4). Using 274 single-copy orthologous genes conserved in these species, we constructed a phylogenetic tree to shed light on the evolutionary history of these compared species. Our analysis revealed that safflower (*C. tinctorius*) and *Helianthus annuus* diverged ~40 million years ago (MYA), while *Helianthus annuus* and *Artemisia annua* (or *C. nankingense*) diverged ~32 MYA (as estimated), which is generally consistent with taxonomic classification and fossil evidence (Fig. 2A). Furthermore, we utilized CAFÉ to examine the evolution and expansion of safflower gene families, indicating that 11 984 gene families underwent contraction, while 1824 gene families underwent expansion (Fig. 2A). To investigate the WGD event, we used the distribution of synonymous substitution rates (*K*_s_) of all gene pairs examined in each chromosome segment. We detected two peaks at roughly 2 and 0.25 in the safflower genome, which corresponded to the core eudicot γ-triplication event and a safflower-specific WGD event during evolution, respectively (Fig. 2B).

For detailed analysis, we selected the genomes of *Helianthus annuus* and *Vitis vinifera* for synteny mapping in plant molecular biology. Based on the conserved gene order, we identified 1922 syntenic blocks between safflower and *H. annuus*, which correspond to 10 374 and 13 349 genes, respectively, in each genome (Fig. 2C, Supplementary Data Fig. S5). Among these blocks, 414 contain >10 genes. On average, each block in the safflower genome includes five genes. Additionally, we detected 1135 collinear blocks common to the safflower and *Vitis vinifera* genomes, corresponding to 9686 and 8434 genes, respectively, in each genome. On average, each block includes 8.5 genes, and 396 blocks have >10 genes. Between safflower and *Arabidopsis thaliana*, we identified 1170 syntenic blocks, which correspond to 6073 and 6575 genes, respectively, in each genome, and on average each block in the safflower genome includes five genes. Large-scale genomic rearrangements, such as duplications, inversions, and translocations, were observed among these four species, indicating extensive genomic shuffling since the divergence of the ancestor of dicotyledons (Fig. 2C, Supplementary Data Fig. S5).

### Metabolites, transcriptome, and integrated analysis of safflower

**Figure 3:**
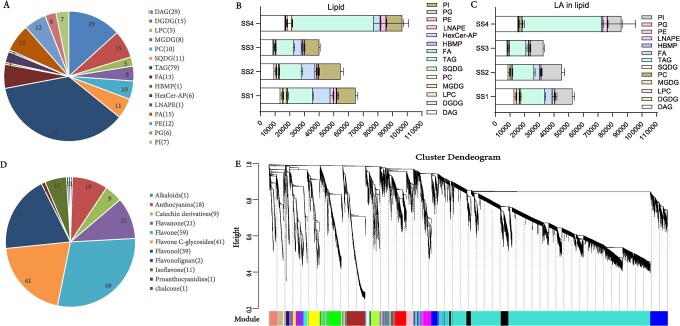
Metabolites of lipid and flavonoids and the transcriptome profile analysis. **A** Lipid composition and the parentheses refer to the number of this ingredient. **B** Lipid composition of seeds at four developmental stages. **C** LA content in each kind of lipid. **D** Flavonoid composition and related numbers. **E** Co-expression network analysis on all the transcriptome data of safflower using WGCNA and 20 modules, including 15 769 genes.

In safflower, the oil content, particularly LA, and flavonoids, particularly HSYA, are two essential traits. To ascertain the lipidome profile during seed development, we detected total lipid metabolites at four different stages (named SS1, SS2, SS3, and SS4) of safflower seeds using UHPLC–MS/MS. We identified 218 compounds and classified them into 13 types (Fig. 3A). which TAGs included 72 compounds, DAGs (Diacylglycerol) included 29 compounds, PCs (phosphatidylcholine) included 15 compounds, and PEs (phosphatidylethanolamine) included 15 compounds (Supplementary Data Table S6). TAG was the dominant lipid (Fig. 3B), and the lipid content increased significantly from SS3 to SS4. We calculated the LA content in different stages by analyzing the composition of different lipid types. In most types of lipid, LA accounted for >90%, except for LPC (Lyso-phosphatidylcholine) (21.39%), PC (70.64%), SQDG (Sulfoquinovosyldiacylglycerol) (81.11%), FA (43.96%), and PG (71.54%). The LA content was highest at the SS4 stage (Fig. 3C). Furthermore, our results showed that LA accounted for the highest percentage among TAGs. We detected flavonoid metabolites using the widely targeted metabolome methods, and identified 203 flavonoid metabolites, which were classified into eight types, including 59 flavones, 41 flavone C-glycosides, 39 flavonols, 20 flavanones, 18 anthocyanins, 11 isoflavones, 2 flavonolignans, 1 quinone chalcone, and 1 alkaloid (Fig. 3D, Supplementary Data Table S7).

To conduct gene expression analysis, we carried out transcriptome sequencing on three different groups: flowers at four stages of development, seeds at four stages of development, and three types of tissue (roots, stems, and leaves). After filtering out low-quality data, we obtained a total of 197.19 Gb of clean data, with each sample achieving Q20 and Q30 scores above 95 and 90%, respectively (Supplementary Data Table S8). By employing Hisat2 and StringTie, we aligned and annotated the data using the safflower genome. The assembled fragments demonstrated high integrity, with a mapping rate ranging from 86.52 to 92.21%, indicating that the sequencing quality was sufficient for subsequent analysis. Utilizing the fragments per kilobase per million (FPKM) method (Supplementary Data Table S9), we analyzed the expression profile and observed notable variations among the different samples (Supplementary Data Table S10). For comprehensive analysis, we employed WGCNA to perform co-expression network analysis on the entire transcriptome data of safflower, resulting in the construction of 20 modules comprising 15 769 genes (Fig. 3E).

**Figure 4:**
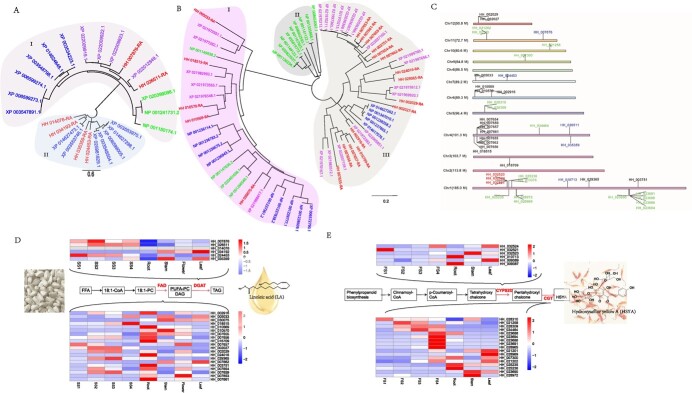
Genetic and expression analysis of the genes for LA and HSYA biosynthesis. **A** Phylogenetic analysis of DGATs. I represents subfamilies of DGAT2. II represents subfamilies of DGAT1. Green color indicates DAGTs from *Z. mays*. Blue indicates DGATs from soybean. Purple indicates DGATs from *H. annuus*. Red indicates DGATs from safflower. **B** Phylogenetic analysis of FAD2. Colors have the same meanings as for DGATs. **C** Chromosome distribution of candidate genes. Green indicates genes for CGTs, red for CYPs, blue for DAGTs, and black for FAD2. **D** Gene expression for DGATs. **E** Gene expression for FAD2. **F** Gene expression for CYPs. **G** Gene expression for CGTs.

To conduct an integrated analysis of the metabolome and transcriptome during four different seed development stages, we established an O2PLS model using all differential genes and lipids. We preliminarily judged the variables with high correlation and weight in different data groups, and screened out genes that affected lipids (Supplementary Data Table S11). Our results showed that HH_016304, annotated as acyl-coenzyme A oxidase 3, peroxisomal-like (A), and HH_015340, annotated as fatty acid omega-hydroxylase, were identified, demonstrating the accuracy of the integrated analysis.

### Genomic analysis of key gene families participating in linoleic acid and hydroxysafflor yellow A biosynthesis

The process of lipid biosynthesis involves several gene families, and one of the crucial enzymes involved is DGAT, which acts as a limiting factor. Previous studies have indicated that different versions of DGAT contribute to the biosynthesis of both saturated and unsaturated fatty acids [26]. To understand the patterns of DGAT evolution better, we specifically extracted individual gene sequences from the safflower genome belonging to the DGAT family, characterized by the Pfam domain PF03982. By using BLAST methodology, we compared these sequences with reported DGAT genes [27] and identified six homologies, which were utilized to construct a phylogenetic tree of the DGAT family (Supplementary Data Fig. S6). The findings revealed a division within DGATs into two subfamilies, namely DGAT1 and DGAT2. Moreover, it was observed that DGAT2s in safflower exhibited close similarity to those found in *H. annuus* (Fig. 4A). Additionally, aside from DGAT, another enzyme, called FAD, is widely known for its role in catalyzing the formation of double bonds in fatty acid chains, resulting in the production of unsaturated fatty acids. Various types of FAD enzymes, such as FAD2, FAD3, FAD4, FAD5, FAD6, FAD7, and FAD8, have been extensively studied [28]. Among these, FAD2 has been identified as a significant enzyme influencing the three primary fatty acids, namely oleic, linoleic, and linolenic acids, in oilseed plants [20]. To investigate the FAD evolution pattern in safflower, we extracted all single FAD domain family gene sequences (PF11960 and PF00487) from the genome and identified a total of 20 FAD2 genes (Supplementary Data Fig. S7). Phylogenetic analysis shows that FAD2 can be divided into three families, and most FADs are related to those of *H. annuus* (Fig. 4B). The location of DGAT and FAD genes in the genome can be viewed in Fig. 4C, with most FAD genes distributed in chromosome 4. To identify which DGAT and FAD isoforms are most likely to participate in LA biosynthesis, we analyzed their expressions. Based on our results, it is likely that HH_026511 and HH_024453 DGAT isoforms play a role in lipid biosynthesis, and HH_029365 FAD isoform may respond to TAG biosynthesis in seeds (Fig. 4D).

HSYA is recognized as a dynamic element, a C-glycosyl compound comprising a 3,4,5-trihydroxycyclohexa-2,5-dien-1-one core with β-d-glucosyl groups attached to positions 2 and 4, alongside a *p*-hydroxycinnamoyl group at position 6. The biosynthesis of HSYA involves two gene families (CYP and UGT), and a schematic diagram of the biosynthesis route can be seen in Supplementary Data Fig. S8. CYP82D2 is known to be an F8H with high substrate specificity for flavone, and in safflower F8H is similar to 4-hydroxycyclohexa of naringenin chalcone, hence its analogs are likely involved in HSYA biosynthesis. We extracted 403 CYP genes from the safflower genome based on the Pfam ID PF00067 and identified seven homologies of CYP82D genes in safflower based on phylogenetic tree analysis (Supplementary Data Fig. S9). The CYP82 in safflower can be divided into three families, all of which are closely related to those in *H. annuus* (Supplementary Data Fig. S10). Another gene family involved in HSYA biosynthesis is UGT, particularly CGT. CGT has been reported to participate in the C-glycosylation of flavonoids [29–31]. We extracted all single UGT-family gene sequences from the safflower genome based on PF00201 and identified 173 genes, of which 19 were putative CGT genes (1 for CGT and 18 for flavone CGTs) (Supplementary Data Fig. S11). Phylogenetic analysis showed that the UGTs in safflower were significantly different from those in other plants, indicating that the HSYA pathway underwent independent evolution. The CGTs in safflower can be divided into four families, all of which are closely related to those in *H. annuus* (Supplementary Data Fig. S12). The location of CYP82 and CGTs in the safflower genome can be viewed in Fig. 4C, with most of the CGTs distributed in chromosome 1. To identify the candidate CYPs and CGTs involved in HSYA biosynthesis, we analyzed their expression patterns. Based on our results, HH_032524 for CYP and HH_034464 for CGT have high expression levels in the flower of safflower (Fig. 4E), indicating that they may be involved in the biosynthesis of HSYA in the flower.

### Significant SNPs associated with gene loci related to key traits of safflower by GWAS analysis

**Figure 5:**
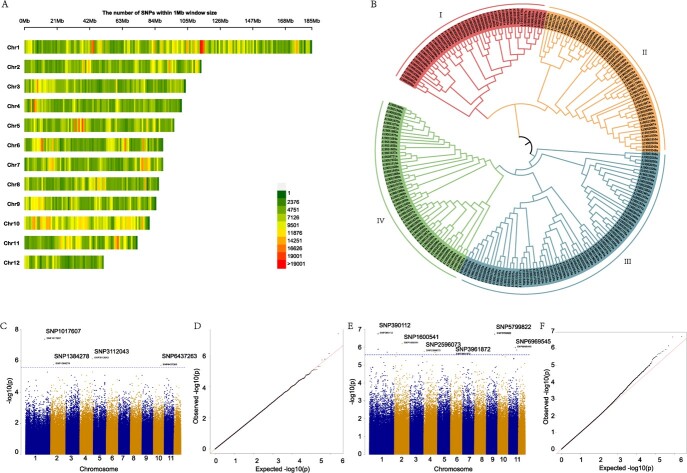
SNP and GWAS analysis with oil content and flower color. **A** SNP distribution among 12 pseudochromosomes of safflower. **B** Phylogenetic tree of the 220 safflower lines. It can be divided into four subgroups. **C** Manhattan plots of assessed morphologic features related to oil content. **D** qq_plot for oil content. **E** Manhattan plots of assessed morphologic features related to flower color. **F** qq_plot for flower color.

We conducted re-sequencing on 220 safflower lines, resulting in the acquisition of a total of 7 402 693 high-quality SNPs. The distribution of these SNPs across the 12 chromosomes is visually represented in Fig. 5A. Employing these SNPs, we performed PCA analysis (Supplementary Data Fig. S13) and constructed a phylogenetic tree encompassing all the lines. To analyze the population structure, ADMIXTURE software was utilized with varied *K* values. Cross-validation error analysis indicated that *K* = 4 yielded the most optimal outcome (Supplementary Data Fig. S14). Notably, the population structure analysis revealed the absence of distinct family differentiation among all samples, thus suggesting their suitability for subsequent GWAS analysis. Furthermore, the phylogenetic tree analysis unveiled four distinct subgroups within the selected population (Fig. 5B), thus confirming the earlier conclusion that *K* = 4 is indeed the optimal outcome obtained via population structure analysis.

**Figure 6:**
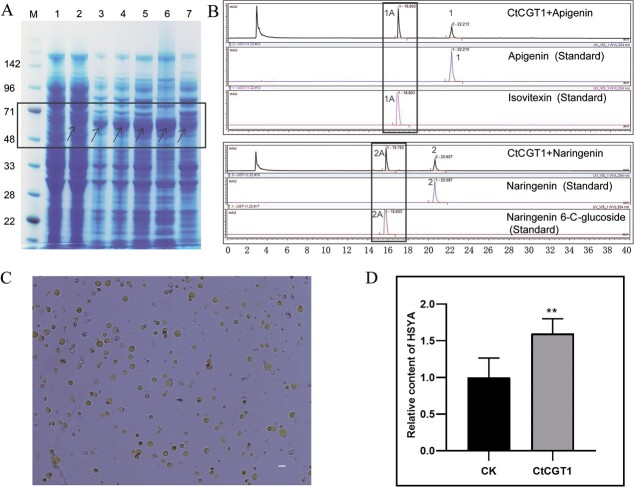
Function analysis of *HH_034464* (*CtCGT1*). **A** Prokaryotic expression of CtCGT1. M is the protein marker. Lane 1, total protein after transferring *CtCGT1* without IPTG induction; lane 2, total protein after transferring *CtCGT1* with IPTG induction; lanes 3–5, total protein after 3, 9, and 16 h with IPTG induction; lane 6, precipitation after ultrasonic fragmentation using the 16 h cultured bacteria with IPTG; lane 7, supernatant after ultrasonic fragmentation using the 16 h cultured bacteria with IPTG. Arrows point to the induced protein. **B** Function analysis of CtCGT1 using crude enzyme. We used apigenin and naringenin as substrates. Isovitexin and naringenin 6-C glycosides were used as the control. CtCGT1 + apigenin and CtCGT1+ naringenin indicates that apigenin and naringenin were added to the crude enzyme. 1 is apigenin, 1A is isovitexin, 2 is naringenin, 2A is naringenin 6-C glycosides. **C** Protoplasts transformed with *CtCGT1*. Bar = 30 μm. **D** Determination of relative content of HSYA. CK is the HSYA content measured after transferring the empty vector. CtCGT1 is the HSYA content measured after transferring *UBI::CtCGT1*. We default the HSYA content in CK value to 1.

We performed GWAS analysis using high-quality SNPs on eight agronomic traits (BN, ball number; BH, branch height; BS, bract spine; FBN, first branch number; FC, flower color; OC, oil content; PH, plant height; SD, stem diameter). All trait data records can be found in Supplementary Data Table S12. To ensure the accuracy of the analysis, PLINK software was utilized to remove linked SNPs and eliminate interference from linked loci in the statistical model. This resulted in 1 239 895 valid SNP sites. To reduce the number of redundant SNPs, we used GEC software to accurately estimate the number of independent tests (Ne) based on linkage disequilibrium. Approximately 123 million SNP markers in total were analyzed across the eight traits, and significantly related SNPs were found for all eight traits in safflower. Among these significantly related SNPs, there were 4 and 12 SNPs significantly related to OC and FC traits, respectively (Fig. 5C–F). The 4 SNP markers related to OC were distributed across four different chromosomes, while the 12 SNP markers related to FC were distributed across six different chromosomes. For the SNP marker related to FC, HH_018127 was annotated as YABBY transcription factor CDM51. YABBY proteins are a group of plant-specific transcription factors involved in flower development [32, 33]. Additionally, HH_035307 is a member of cytochrome_CBB3, and both genes may participate in flavonoid biosynthesis in flowers. As for SNPs related to OC, two SNPs (SNP1017607 and SNP6437263) lead to more oil content in safflower (>26% oil content) (Supplementary Data Table S13). A new gene (HH_014546) was identified. It should be noted that HH_014546 also showed significant impact on lipid biosynthesis based on the integrated analysis of metabolites and transcriptomes of safflower seed development. All of the related SNP markers provide candidate genes for further research. Detailed information can be found in Supplementary Data Table S14.

### *HH_034464* is involved in the biosynthesis of hydroxysafflor yellow A in safflower

We conducted functional verification on the screened candidate genes and specifically selected *HH_034464* (named *CtCGT1*) through integrative omics analysis. To investigate *CtCGT1*, we employed a widely used prokaryotic expression system for glycosyltransferase identification [34]. First, we induced the expression of CtCGT1 using IPTG (Fig. 6A). The coding sequence region of *CtCGT1* is 1320 bp and the predicted size of CtCGT1 is 48.7 kDa. The tag on the expression vector is ~10 kDa, indicating that the induced CtCGT1 with tag should be ~60 kDa. The actual size of the induced protein on the SDS–PAGE is observed to be between and 71 kDa (Fig. 6A, lanes 2–7), which indicated that CtCGT1 was successfully induced. Furthermore, CtCGT1 can be detected in the supernatant (Fig. 6A, lane 7), indicating its solubility. Using the crude enzyme solution, we examined CtCGT1 activity towards apigenin and naringenin, where only one new product emerged (Fig. 6B). The new components generated correspondingly have identical retention times with apigenin 6-C glycosides (isovitexin) and naringenin 6-C glycosides (Fig. 6B). Mass spectrometry analysis also reveals that the MS2 of the product of CtCGT1 and apigenin are the same as that of isovitexin (Supplementary Data Fig. S16A). Similarly, the MS2 of the products of CtCGT1 and naringenin are identical to those of naringenin 6-C glycosides (Supplementary Data Fig. S16B). This attests that the products are isovitexin and naringenin 6-C glycosides, which confirmed that the glycosylation of CtCGT1 occurred at the 6-C position of the flavonoids.

In our previous study [35], safflower protoplasts were successfully prepared and utilized to investigate the regulation of flavonoid biosynthesis in flowers. To achieve this, we overexpressed *CtCGT1* in protoplasts (Fig. 6C) and subsequently measured the HSYA content. The results showed that the protoplasts containing CtCGT1 exhibited increased HSYA content compared with the control group with an empty vector (Fig. 6D). These findings suggest the involvement of CtCGT1 in HSYA biosynthesis.

## Discussion

Compared with the versatile applications of safflower, this crop remains underappreciated. Acquiring a complete and highly annotated genome sequence is crucial for identifying gene functions associated with important traits and for plant breeding purposes. In a previous study by Bowers *et al*., a draft genome assembly of safflower was generated, covering approximately two-thirds (866 million base pairs) of the estimated 1.35-Gb genome. Unfortunately, the contig N50 size was only 368 bp, and the scaffold N50 size was 1976 bp, indicating low-quality genome information due to limited sequencing technology and sequencing depth [36]. Wu *et al*. later published a chromosome-level assembly of safflower using PacBio single-molecule real-time (SMRT) and Hi-C sequencing for the ‘Anhui-1’ variety, which possesses high LA content [15]. However, it should be noted that a single reference genome may not fully represent all genes within a species. ‘Chuanhonghua 1’ safflower is an ancient variety widely cultivated in Sichuan, China, with medicinal and culinary uses. Researchers performed *de novo* sequencing of its genome and reported a successful mapping rate of 92.36%, with 97.2% of mapped genes being complete (89.79% of the total) and 91.42% of complete genes having a single-copy mapping (84.44% of the total) (Supplementary Data Table S3). Furthermore, when comparing our genome with the published ‘Anhui-1’ genomes by Wu *et al*. [15], we have achieved some superior indicators; for example, our annotated genes outnumber the published genomes, with a count of 39 809 compared with their 33 343. These findings demonstrate the integrity, continuity, and precision of the final sequence assembly.

In this study, we analyzed the metabolite characteristics of lipids and flavonoids. Typically, GC–MS is used to detect lipid compounds, specifically to identify different fatty acid types [37, 38]. However, it does not represent the original lipid composition. Here, we used UHPLC–MS to detect lipidomics. By analyzing the different lipid types, we calculated the LA content during seed development. Most lipids contained >90% LA, with linoleic acid exhibiting the highest increase at the SS4 stage (Fig. 4C). Additionally, LA from TAG had the highest percentage, and PC accounted for the majority of LA (70.64%). Previous reports suggest that unsaturated fatty acids come through the PC pool and PC pathway [39]. Therefore, the results of our lipid compound analysis imply that the LA in safflower seeds may also be sourced from the PC pool.

In safflower seeds, the predominant component of oil is PUFA, with LA constituting ~76% of the total oil, α-LA accounting for ~0.04%, and no presence of γ-LA in the oil [40]. FAB2, FAD2, and FAD3 are essential desaturases involved in the biosynthesis of LA and OA. FAD2 plays a critical role in converting OA to LA, whereas FAD3 regulates the content of linolenic acid (18:3) through the desaturation of LA (18:2) [20]. FAD2 was previously analyzed in a study [15], but it is worth noting that DGAT also plays a significant role in controlling carbon flux towards TAG biosynthesis in the Kennedy pathway, functioning as a rate-limiting and key enzyme [22]. Analyzing the genome of DGAT will provide insights into the high LA content in safflower seed oil. In plants, DGAT1 and DGAT2 are two major forms of DGAT with distinct functions in TAG biosynthesis. Previous studies have indicated that DGAT1 plays a primary role in TAG production in oilseeds and fruits [41], while DGAT2 is crucial for TAG biosynthesis by accumulating unusual fatty acids [26]. As for DGAT expression, it is likely that HH_026511 is involved in lipid biosynthesis in safflower seeds. Further research can verify the function of these genes in HSYA biosynthesis based on this genetic information.

Currently, there is a comprehensive understanding of the fundamental flavonoid biosynthetic pathway [23, 24]. However, no reported gene is known to be involved in the biosynthesis of HSYA and its derivative, anhydrosafflor yellow B, which are specific compounds found in safflower. Considering the structure of HSYA, it is possible that two gene families, CYPs and UGTs, play a role in the biosynthesis pathway. Several UGTs have been reported in other plants, including citrus plants for C-glucosyl flavonoids [29], *Glycyrrhiza glabra* [42], and *Medicago truncatula* for O-glucosyl flavonoids [43]. Within our study, we identified 28 putative CGTs from the CGT708 gene family. Our findings suggest that one gene is closely associated with chalcone CGT while the remaining 27 genes are linked to flavone CGT. The gene related to chalcone CGT is likely responsible for the biosynthesis of 3′ CGT tetrahydroxychalcone. Similarly, one other gene among the other 27 genes associated with flavone CGT probably contributes to the biosynthesis of 5′ CGT tetrahydroxychalcone. Interestingly, the results of prokaryotic expression and protoplast experiments showed that HH_034464 (*CtCGT1*) (one of the 27 genes screened) belongs to the gene family of 6-glycosyltransferases. This validates the accuracy of our screening results.

The challenges associated with traditional genetic approaches have led to the development of genomic resources for addressing issues related to plant cultivation and accelerating the discovery of significant trait genes. GWAS present an enticing alternative for identifying loci that control crucial traits and have been widely adopted for identifying candidate genes for specific traits in economically important plants [44–46], with SNP markers benefiting molecular plant breeding. High-quality genome assemblies have a crucial role in facilitating mapping in GWAS. In our research, we established a high-quality, chromosome-scale reference genome of safflower using an integrated strategy. Additionally, for the first time, we collected 220 safflower lines from diverse global genetic backgrounds, which is critical to obtaining reliable results in GWAS analyses. Together, these developments provide an excellent foundation for GWAS analyses of safflower.

Apart from the 4 and 12 SNPs that are significantly associated with OC and FC traits, respectively, we identified 5 SNPs linked to BH traits, 42 SNPs linked to BN traits, 104 SNPs linked to BS traits, 51 SNPs linked to FBN traits, 84 SNPs linked to PH traits, and 17 SNPs linked to SD traits (Supplementary Data Fig. S15 and Supplementary Data Table S14). There remain several candidate genes that require further validation. Nevertheless, all the results offer valuable data for functional gene analysis, which will be instrumental for advancing safflower breeding for industrial and medicinal purposes.

## Materials and methods

### Materials

We employed ‘Chuanhonghua 1’, a safflower variety, for genome sequencing. This variety is cultivated at the medicinal botanical garden located on the Wenjiang Campus of Chengdu University of Traditional Chinese Medicine. Our laboratory has maintained this variety through self-inbreeding for >10 years. To detect flavonoids, we utilized fully bloomed flowers that were ~3 days old after pollination. For lipid analysis, we examined seeds at different stages of development. To analyze the expression profiles, we collected roots, stems, leaves, and flowers 3 days after pollination and seeds at four different developmental stages. To extract total RNA, we followed the manufacturer’s instructions and used Trizol (Invitrogen, CA, USA). Through collaboration with a cooperative unit, we obtained 220 safflower lines from various parts of the world, each line being self-inbred and characterized. In 2020, we evaluated the agronomic traits of these safflower lines.

### Genome sequencing, assembly, and annotation

We employed the CTAB method for the extraction of genomic DNA from safflower leaves. To create a nanopore library, we produced 10-kb fragments and acquired 29.71 Mb of nanopore reads, averaging 3.75 kb in length, using the Nanopore GridION system. Additionally, we generated a paired-end Illumina library with the Illumina HiSeq X Ten instrument. For Hi-C sequencing, we utilized freshly harvested safflower leaves. The resulting products were enriched, physically sheared to 350 bp, and subjected to DNA purification. Biotin was removed from unligated ends. To estimate the genome size of safflower, we used Illumina clean reads with the jellyfish tool (−m 21 -s 1000000000) v2.3.0 [47]. Initial nanopore reads were generated using Canu (corOutCoverage = 80) v1.71 [48], and the contigs were subsequently improved using Illumina clean reads using NextPolish v1.3.1 [49]. LACHESIS v1 was employed to connect contigs based on Hi-C data, with default parameters. The reassembled contigs were refined by using Create Scaffolded Fasta.pl. Gene prediction was performed using EvidenceModeler v1.1.1, which combines *ab initio* gene prediction tools such as Augustus v3.3.1, Gene Mark-ES v4.3.3, and SNAP v20131129, as well as homology gene prediction tools such as BLAST v2.2.28 and GeneWise v2.2.0. The reference used for homology gene prediction was *A. thaliana* data. To generate and train gene models, we utilized the full­length ISO­seq pipeline with PacBio, and RNA­seq assemblies were produced by Trinity v2.8.5 [50]. The assembly and gene prediction were evaluated using BUSCO v3.0.0 [51]. RepeatMasker v4.0.7 was employed for the annotation of repetitive elements. Furthermore, we employed RepeatModeler v1.0.8 [52] to construct a *de novo* database, with REPBASE serving as the reference for comparison.

### Genome evolution analysis and construction of the phylogenetic tree

We employed Hcluster_sg v0.5.1 to group single-copy orthologous genes, followed by alignment using MUSCLE v3.4. Maximum likelihood phylogenetic trees were constructed using PhyML v3.0 9 [53], and divergence times were inferred using the R8s package v1.81. Calibration times were obtained from the TimeTree website (https://timetree.org). Gene family expansion/contraction was analyzed using CAFÉ v4.2.1, and syntenic comparisons were performed using MCscan v0.9.6 [54] with default parameters.

### Lipid extraction and UHPLC–MS/MS analysis

We employed a technique based on our prior investigation [65] for extracting lipids. About 30 mg of the specimen was measured, and 100 μl of a whole-lipid internal standard (10 μg/ml) was inserted. The mixture was vigorously stirred with 2 ml of methanol at −20°C for 12 h, followed by the inclusion of 2 ml of methylene chloride and subsequent stirring for 1 h. After adding 2 ml of methylene chloride and 1.6 ml of ultrapure water, the solution was centrifuged. The upper layer was discarded, while the lower layer was combined with the lower layer from a second extraction. The amalgamation was then evaporated using a nitrogen blow, reconstituted in 1 ml of isopropyl alcohol, filtered through an organic membrane filter with a pore size of 0.22 μm, and subjected to LC–MS/MS analysis. The UHPLC–MS/MS assessments were carried out at a facility in Beijing, China, using a Vanquish UHPLC system (Thermo Fisher, Germany) connected to an Orbitrap Q Exactive™ HF mass spectrometer (Thermo Fisher, Germany). The solvent gradient and mass spectrometer settings were optimized to ensure precise analysis.

### Flavonoid extraction and UHPLC–MS/MS analysis

We employed a technique based on our prior investigation [66] to extract the flowers. The freeze-dried flowers were crushed using a mixer mill along with a zirconia bead, followed by an overnight extraction at 4°C using 70% aqueous methanol. Subsequently, the extracts were filtered through an SCAA-104 filter featuring a pore size of 0.22 μm, before conducting LC–MS analysis. The UHPLC column utilized was a Waters ACQUITY UHPLC HSS T3 C18, employing a gradient program and optimized solvent system for the analysis. The effluent was connected to an ESI-triple quadrupole-linear ion trap (Q TRAP)-MS instrument, with LIT and QQQ scans acquired in positive ion mode utilizing MRM experiments. Instrument tuning and mass calibration were executed using polypropylene glycol solutions in QQQ modes. DP and CE were optimized for each MRM transition, while a specific set of MRM transitions was monitored for each time period in accordance with the eluted metabolites during the said period.

### Metabolite data analysis

Unsupervised PCA analysis was carried out utilizing the ‘prcomp’ function from www.r-project.org, a popular R statistics library. To standardize the data, prior to conducting unsupervised PCA, unit variance scaling was implemented. Results of hierarchical cluster analysis (HCA) for both metabolites and samples were portrayed through heat maps containing dendrograms. Pearson correlation coefficients (PCCs), expressing the correlation between samples, were obtained using the ‘cor’ function in R and represented in the form of a heat map. Both the HCA and the PCC analysis were performed using the heat map package in R. In the HCA, normalized signal intensities of metabolites (using unit variance scaling) were visualized through a spectrum of colors. Differential metabolites were selected based on VIP values ≥1 and absolute log_2_(fold change) ≥1. VIP values were derived from the OPLS-DA outcome, which includes score plots and permutation plots created using the R package MetaboAnalystR. Prior to running OPLS-DA, Pareto scaling was applied to the data, while overfitting was addressed by conducting a permutation test with 200 permutations.

### RNA sequencing and data analysis

The Illumina HiSeq platform was used to conduct RNA sequencing. High-quality, clean reads were obtained via filtering and trimming using the Trimmomatic package. To align the reads to the safflower genome, HISAT2 was utilized, and StringTie was used to generate transcripts and their expressions. Transcript abundances were quantified via the FPKM method. A threshold of fold change ≥1 and an adjusted *P*-value ≤.01 were set for significance testing. For Gene Ontology (GO) analysis, functional classifications were employed, while Kyoto Encyclopedia of Genes and Genomes (KEGG) classification maps were acquired by KEGG retrieval.

### Screening of gene families participating in linoleic acid and hydroxysafflor yellow A biosynthesis

Homologous genes involved in the biosynthesis of HSYA, including CYP82 and UGTs, as well as genes involved in LA biosynthesis, such as FADs and DGATs, were identified in the safflower genome based on Pfam annotations. The FAD gene family was referred to in the research conducted by Liu *et al*. [55], the CYP gene family in the research of Zhao *et al*. [56], the CGT gene family in the research by Ito *et al*. [29], and the DGAT family in the research by Turchetto-Zolet *et al*. [27]. The conserved domains were verified by submitting all sequences to the website http://plants.ensembl.org/index.html. The CYP82 gene family corresponds to PF00067, while the CGT family corresponds to PF00201. The DGAT family corresponds to PF03982, which features the lysophospholipid acyltransferase (LPLAT) domain, which plays a critical role in glycerophospholipid biosynthesis. The FAD family corresponds to PF11960 and PF00487 and contains PLN02498, involved in ω-3 fatty acid desaturase, and another critical domain, PLN02505, which responds to ω-6 fatty acid desaturase. Additionally, most FAD genes possess the membrane-FADS-like domain, which is a non-heme, iron-containing, oxygen-dependent enzyme involved in the regioselective introduction of double bonds into fatty acyl aliphatic chains. These enzymes play a vital role in maintaining the proper structure and functioning of biological membranes. Finally, the maximum likelihood method was employed in Geneious 11 software to construct the phylogenetic tree for each gene family with a bootstrap value of 1000.

### GWAS analysis

We performed re-sequencing on a set of 220 safflower lines using the GATK 4.0 pipeline [57] to identify mutations and filter SNP sites. Specifically, filtering criteria recommended by GATK, such as Variant Filtration —filter-name FS and —filter ‘FS > 30.0’, —filter-name DP and —filter ‘DP < 5’, and —filter-name MQ and —filter ‘MQ <= 50.0’, were employed. Additional filtering using vcftools [58] was performed to implement a set of specific standards, including maf < 0.05, filtering loci with a deletion rate >0.1, and retaining only biallelic loci to ensure the quality of subsequent analysis. We used Beagle [59] to perform site error correction and genotype filling for all retained sites. PCA was implemented using PLINK 1.90 [60], analyzing 10 principal components and plotting the first two components. Population structure was analyzed using ADMIXTURE software [61] by performing 1000 iterations from *K* = 2 to *K* = 8, and selecting the best *K* using cross-validation errors. Phylogenetic analysis was performed with Treebest software [62] and data visualization was achieved using iTOL software (https://itol.embl.de/). The ADMIXTURE analysis revealed that the plant materials did not display any significant family differentiation, which led us to GWAS analysis. To reduce the effect of linkage site on the statistical model, specific filtering criteria of —indep-pairwise 100 10 0.2 was employed with PLINK software. EMMAX software [61] was used to conduct GWAS tests correlating eight different traits (BN, ball number; BH, branch height; BS, bract spine; FBN, first branch number; FC, flower color; OC, oil content; PH, plant height; SD, stem diameter) with a correlation matrix (*K* model). All trait data records can be found in Supplementary Data Table S12. Effective independent tests (Ne) were estimated using GEC software [62] to reduce the redundancy of SNP information, and *P* < 2.72e−6 (*P* = 1/Ne, Ne = 367 544) was set as the threshold for significant associations. Finally, SNP sites were annotated using ANNOVAR software [63].

### Recombinant protein expression and product detection for enzyme reaction

The *CtCGT1* gene was inserted into the *pET32a* vector (Thermo Fisher, USA) to enable prokaryotic expression. The resulting recombinant vectors, referred to as pET32a-CtCGTs, were then introduced into the bacteria (BL21, DE3) strain) using the heat-shock method. Culture of the BL21 cells took place in 1 l of LB medium supplemented with 50 μg/ml ampicillin at 37°C, with shaking at 200 rpm. The cells were allowed to grow until the OD600 reached 0.5. Induction of the cells occurred by adding 120 μM of IPTG after cooling the flask on ice for 10 min. To establish the optimal induction time, a series of gradient experiments spanning from 3 to 16 h were conducted, eventually leading to the adoption of overnight induction for subsequent experiments. The cells were then incubated for 16 h at 16°C and 200 rpm, followed by collection and resuspension of the cell pellet in 30 ml of lysis buffer (25 mM HEPES pH 8, 500 mM NaCl, 5 mM imidazole). Sonication on ice was employed for cell lysis, and the resulting mixture was subsequently subjected to centrifugation at 6517 g and 4°C for 15 min to remove the supernatant. Finally, the resulting cell pellet was utilized for the initial detection of enzyme activity. To assess the activity of CtCGT1, crude enzyme solution obtained from prokaryotic expression was employed. The reaction products were analyzed using UHPLC in combination with corresponding glycoside standards. The reaction conditions comprised a 1 mM acceptor substrate, 2 mM UDP glucose, 10 μM purified recombinant protein, and a 50 mM Na_2_HPO_4_-NaH_2_PO_4_ buffer (pH 8) in a 100-μl reaction volume. The reaction was allowed to proceed for 5 min at 25°C. Subsequently, 200 μl of precooled methanol was swiftly added to the mixture for efficient mixing and homogenization. After centrifugation at 12 000 rpm for 15 min, the supernatant was collected for further analysis. For UHPLC analysis, an Agilent Eclipse Plus C18 column (150 mm × 3.0 mm, 1.8 μm) was utilized, with the column temperature set at 30°C. The mobile phase consisted of pure water with 0.1% formic acid (A) and acetonitrile (B). A gradient elution process was employed, starting at 5% phase B and reaching 95% phase B over a period of 0–40 min, at a flow rate of 0.2 ml/min.

## Acknowledgements

This project is supported by grants from the National Natural Science Foundation of China (82274039, U19A2010), the Key R&D Plan of Science and Technology Department of Sichuan Province (2021YFYZ0012-5, 2020YFN0152), the Sichuan Provincial Central Guiding Local Science and Technology Development Special Project (2020ZYD058), the Innovation Team and Talents Cultivation Program of National Administration of Traditional Chinese Medicine (ZYYCXTD-D-202209), and the Xinglin Talent Program of Chengdu University of TCM (0300510007). We thank Genebang (www.genebang.com) for providing assistance in data analysis, and AJE (www.aje.cn) for polishing the article.

## Author contributions

J.C., J.X., and J.P. conceived the study. J.C. and J.P. provided funding. X.H., R.W., D.J., Q.L., Z.H., and P.W. prepared the tissue samples for sequencing. J.C., S.G., X.Y., X.L., C.R., S.C., and J.X. led the bioinformatics analyses. S.G., X.Y., and X.L. conducted transcriptome sequencing and analysis. C.R., S.D., and C.C. conducted metabolome analysis. S.G., X.Y., and X.L. constructed the database. X..H, R.W., D.J., Q.L., Z.H., and P.W. recorded the agronomic characters and determination of oil content. J.C. and S.G. drew the figures. J.C. and S.G. drafted the manuscript. X.H., R.W., D.J., S.C., J.X., and J.P. contributed to the writing.

## Data availability

The safflower genome sequence and annotation files have been uploaded to NGDC (National Genome Science Data Center); the project number is PRJCA009936 and the genome number is GWHBJIR00000000. All the RNA data have been uploaded to the Sequence Read Archive (SRA) (http://www.ncbi.nlm.nih.gov/) with accession number SUB13799758. The metabolic data of both seeds and flowers are listed as additional supporting information and can be found in the online version of this article.

## Conflict of interest

The authors declare no conflict of interest.

## Supplementary Material

Supplementary_figures-used_uhad197

Supplementary_Tables-used-revised_uhad197
