# Supplementary material for: Whole-genome and genome-wide association studies improve key agricultural traits of safflower for industrial and medicinal use
Source: Hortic Res. 2023 Sep 29;10(11):uhad197. doi: 10.1093/hr/uhad197 (PMC10673658; doi:10.1093/hr/uhad197)
Supplement: Supplementary_figures-used_uhad197 [file supplementary_figures-used_uhad197.zip › Supplementary_figures-used_uhad197.docx]

**Supplementary figures for the manuscript**

Figure S1 The pipeline for safflower genome assembly in this study

Figure S2 The k-mer analysis of safflower genome

Figure S3 Safflower Hi-C connection heat map

Figure S4 Orthologous genes found in different plant species. Core-multi: genes have orthologues in all other species and might have paralogues in species within one family. Core-single copy: genes have orthologues in all other species and no other paralogues in this species within one family. Unique: genes for which only one family contains genes of this species. Other orthologues: genes are not included in the other mentioned categories. Unclustered genes: genes that are unclustered into any family

Figure S5 Dot-plots showing synteny of the safflower genome with the *H. annuum*, *V.vinifera* and *A.thaliana* genomes

Figure S6 DGATs in safflower blasted with the reported DGATs. All DGATs in safflower were extracted with the pfam domain PF03982

Figure S7 FADs in safflower blasted with the reported FADs. All FADs in safflower were extracted with the pfam domain PF11960 and PF00487

Figure S8 The speculative pathway of HSYA

Figure S9 CYPs in safflower blasted with the reported CYP82. All CYPs in safflower were extracted with the pfam domainthe pfam domain PF00067

Figure S10 Phylogenetic analysis of CYPs in safflower with the homologies gene of CYP82D from *G. max*, *H. annuus* and *Arabidopsis*

Figure S11 UGTs in safflower blasted with the reported UGTs. All UGTs in safflower were extracted with the pfam domain PF00201

Figure S12 Phylogenetic analysis of UGTs in safflower with the homologies gene of UGTs from *G. max*, *H. annuus* and *Arabidopsis*

Figure S13 PCA analysis by the use of the high-quality SNP loci

Figure S14 The population structure analysis with different K values in ADMIXTURE software

Figure S15 GWAS analysis for the the traits of BH, BN, BS, FBN, PH and SD. A is for the trait of BH (branch height); B is for the trait of BN (ball number); C is for the trait of BS (bract spine); D is for the trait of FBN (first branch number); E is for the trait of PH(Plant height); F is for the trait of SD (stem diameter)

Figure S16 MS2 of products formed by the reaction of CtCGT1 with apigenin and naringenin. A The MS2 of the product formed by the reaction of CtCGT1 with apigenin, which is identical to isovitexin. B The MS2 of the product formed by the reaction of CtCGT1 with naringenin, which is identical to naringenin 6-C glycosides.Figure S1. The pipeline for safflower genome assembly in this study


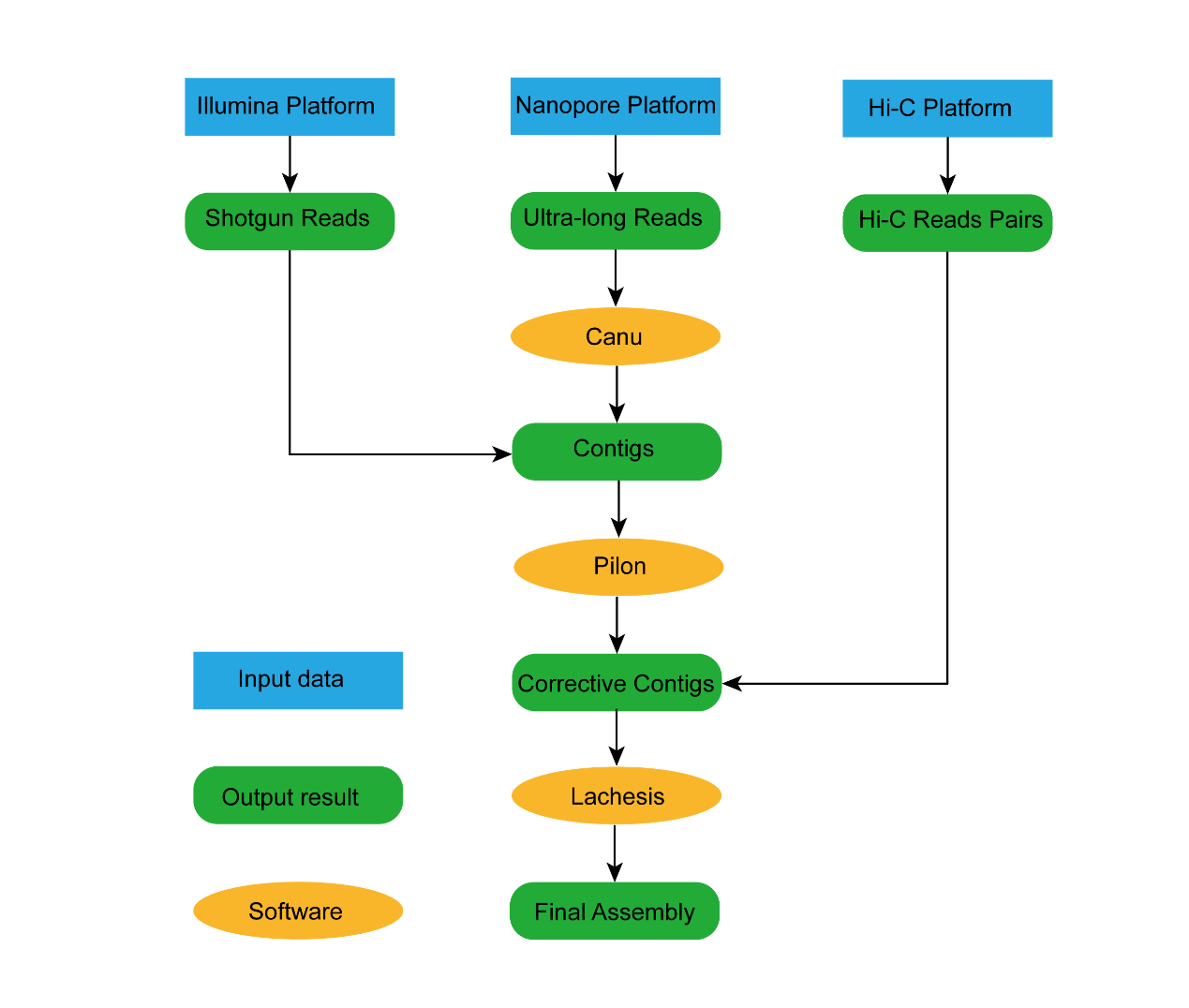


Figure S2. The k-mer analysis of safflower genome

**
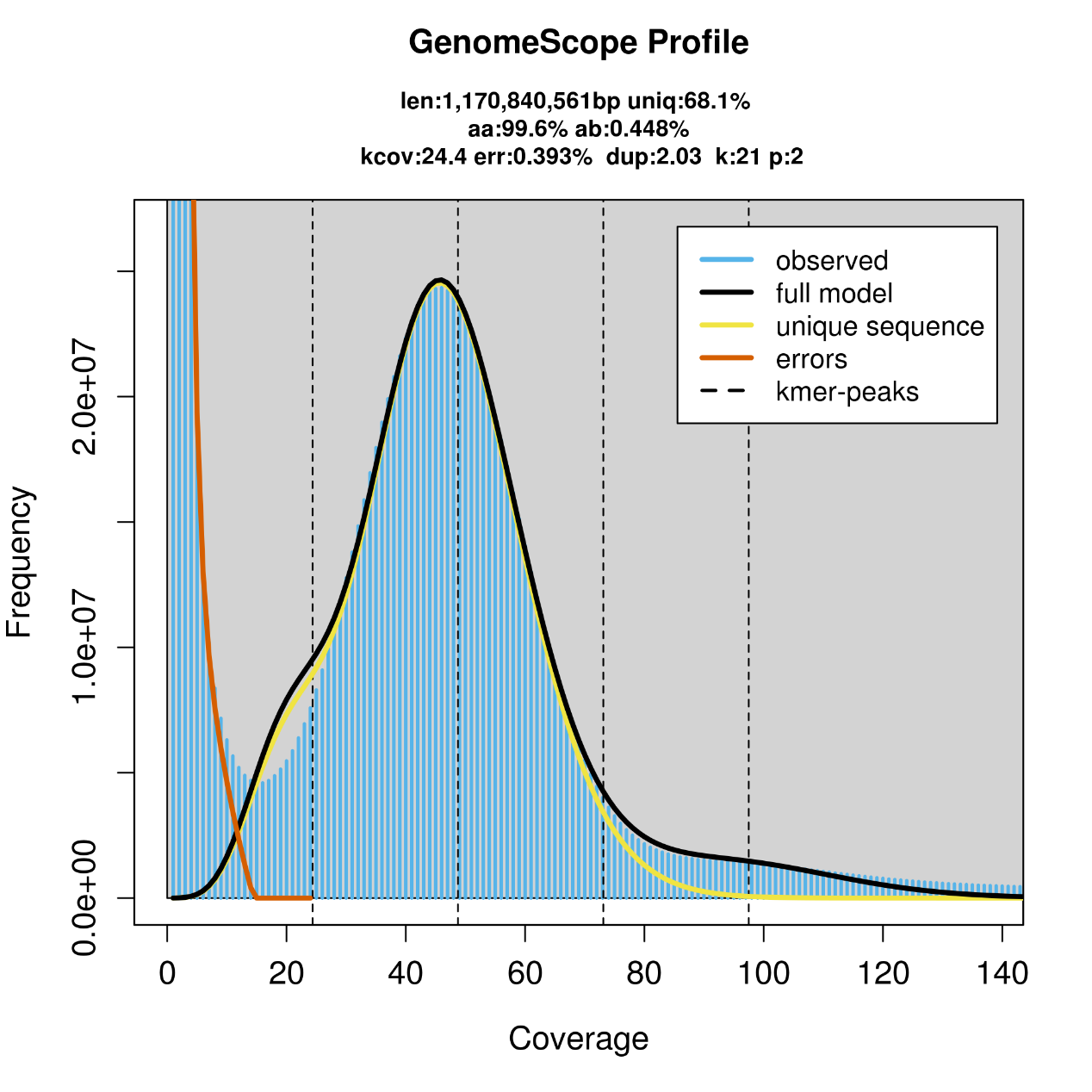
**

Figure S3 Safflower Hi-C connection heat map


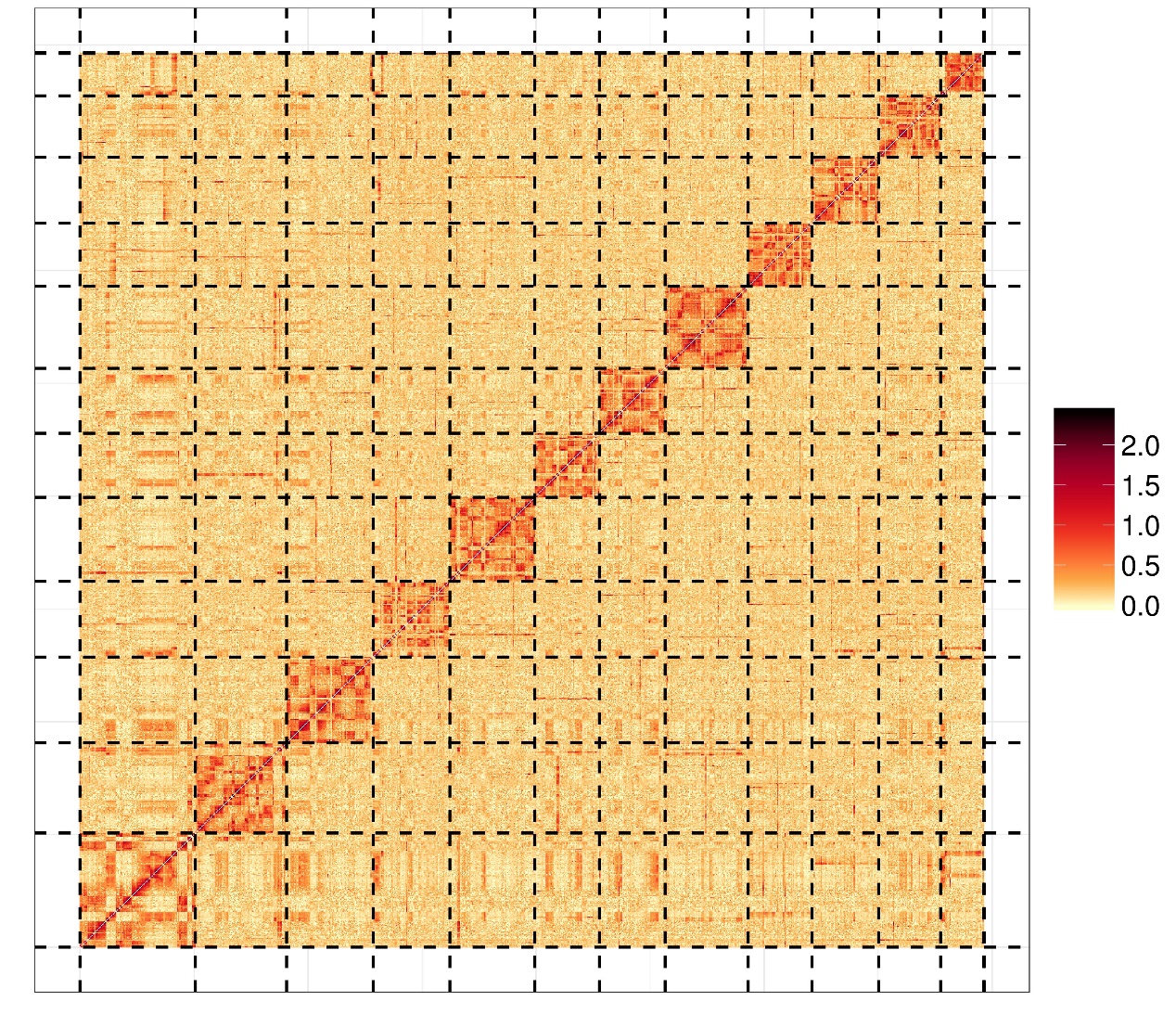


Figure S4 Orthologous genes found in different plant species. Core-multi: genes have orthologues in all other species and might have paralogues in species within one family. Core-single copy: genes have orthologues in all other species and no other paralogues in this species within one family. Unique: genes for which only one family contains genes of this species. Other orthologues: genes are not included in the other mentioned categories. Unclustered genes: genes that are unclustered into any family

**
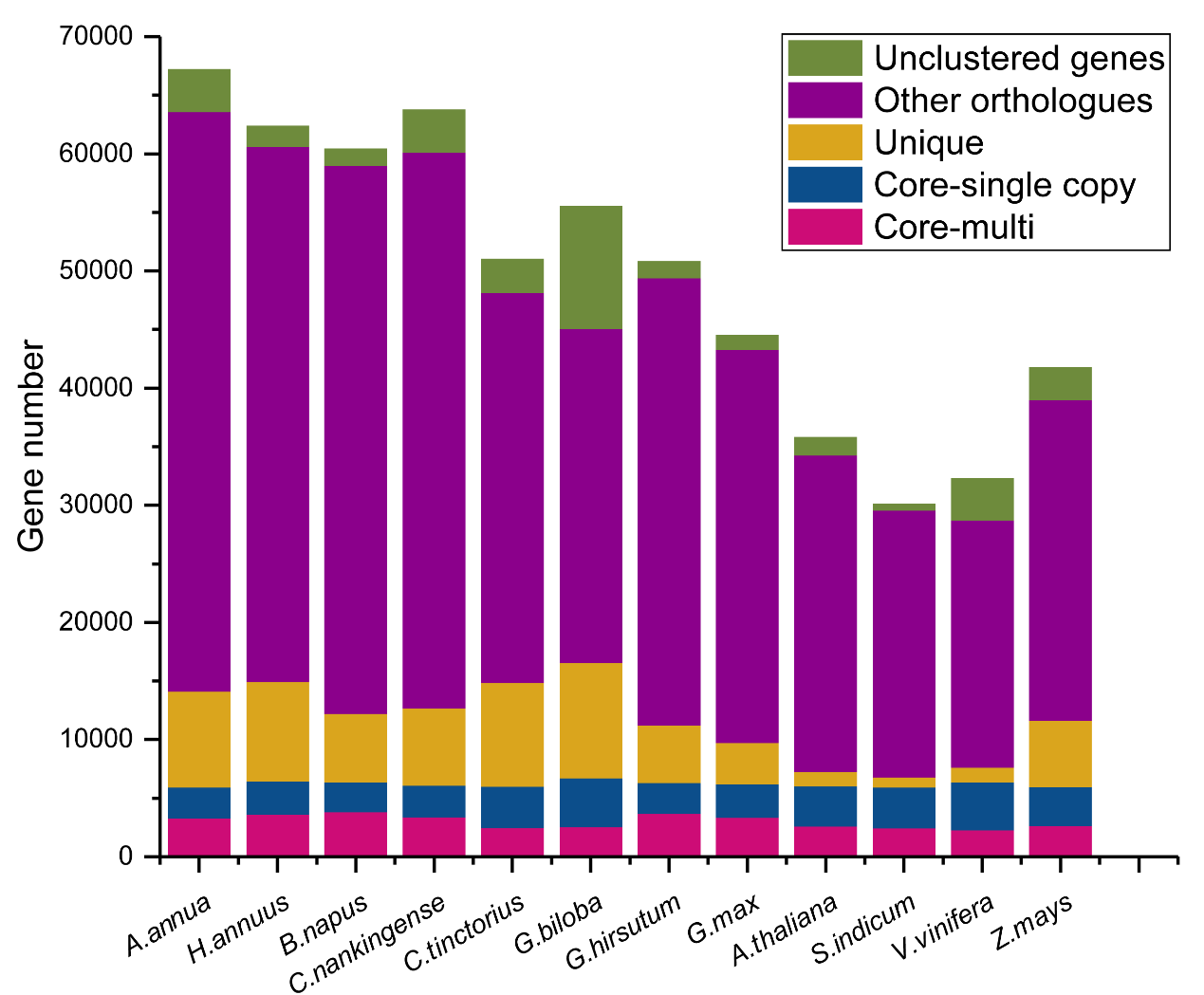
**

Figure S5 Dot-plots showing synteny of the safflower genome with the *H. annuum*, *V.vinifera* and *A.thaliana* genomes

**
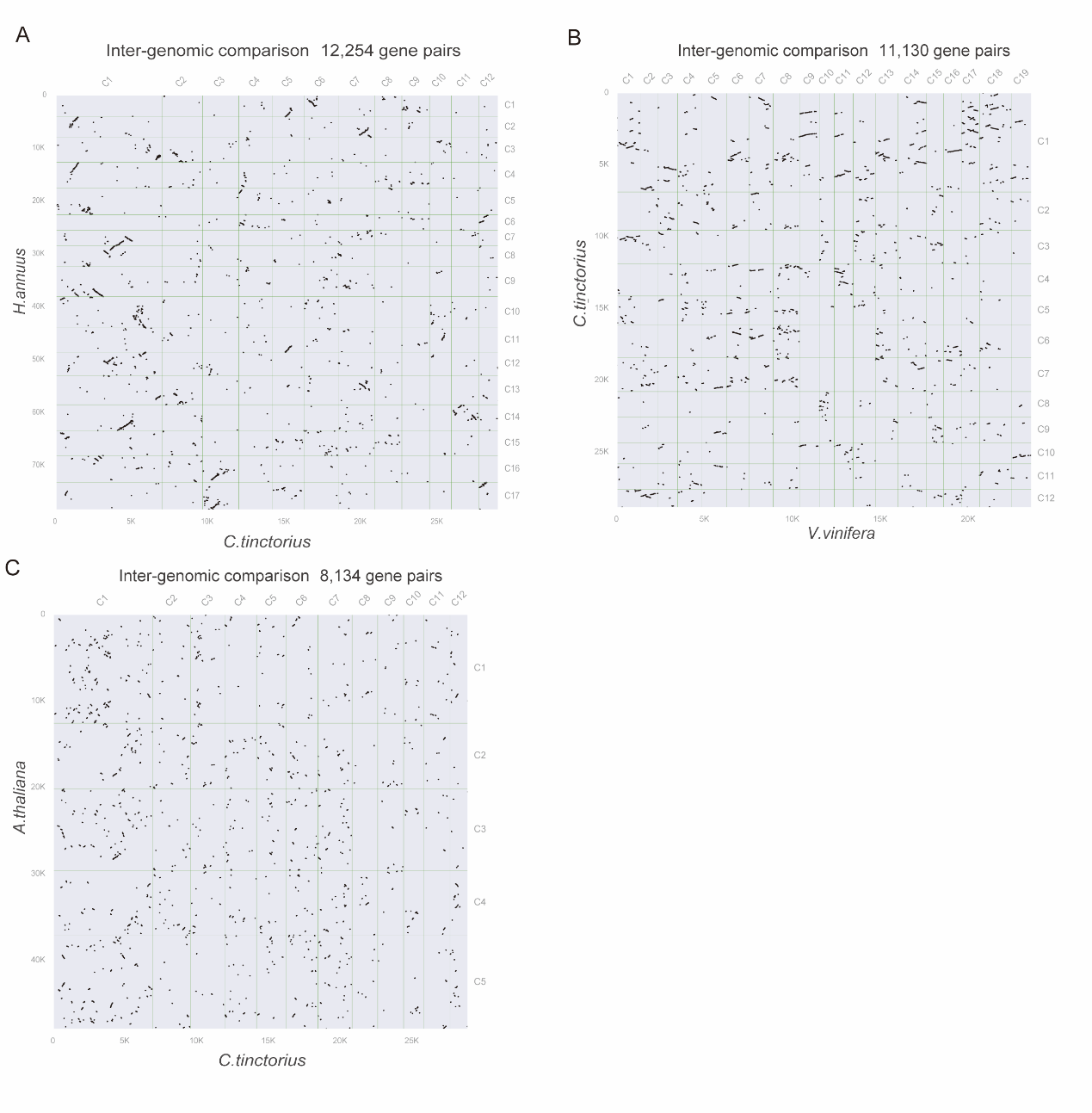
**

Figure S6 DGATs in safflower blasted with the reported DGATs. All DGATs in safflower were extracted with the pfam domain PF03982


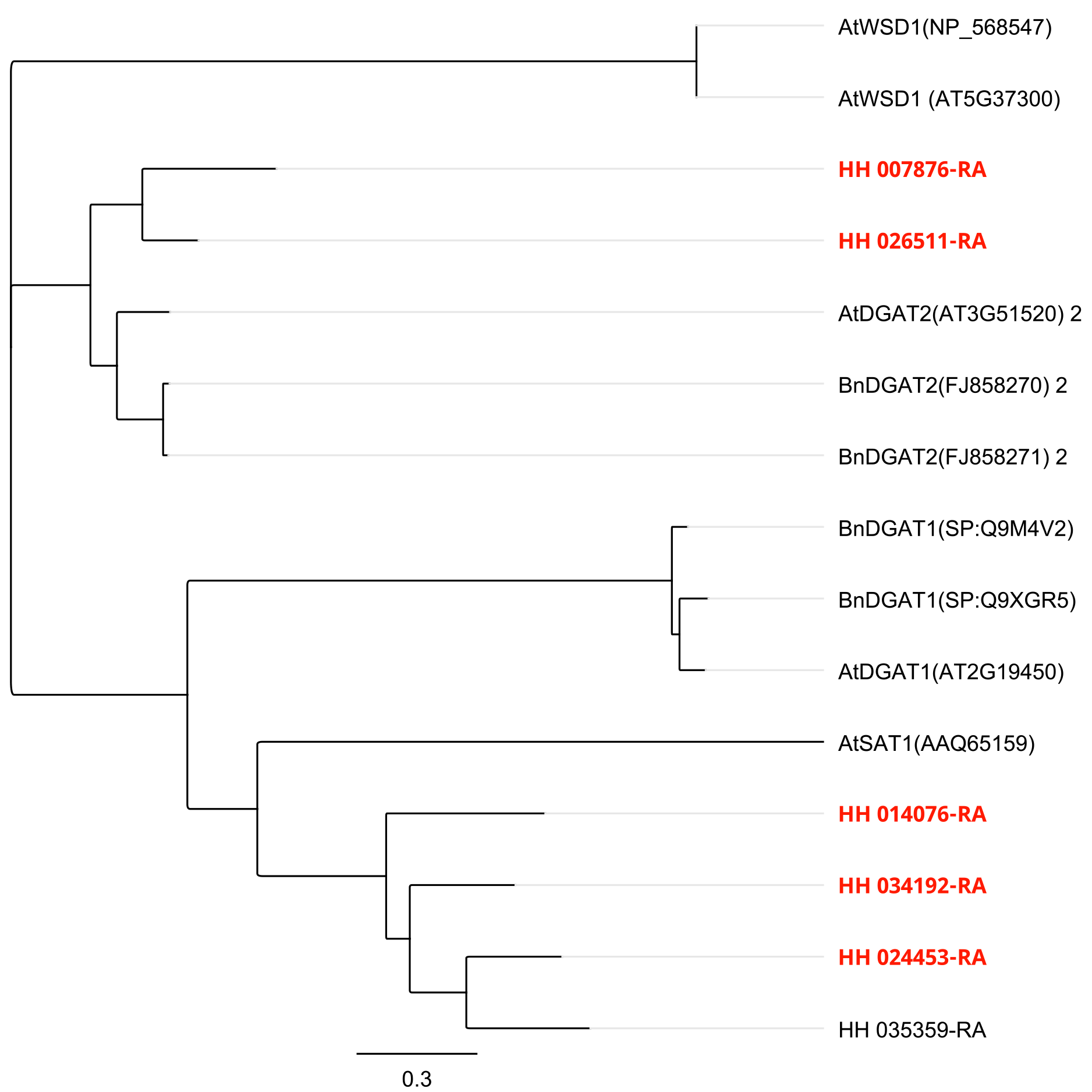


Figure S7 FADs in safflower blasted with the reported FADs. All FADs in safflower were extracted with the pfam domain PF11960 and PF00487


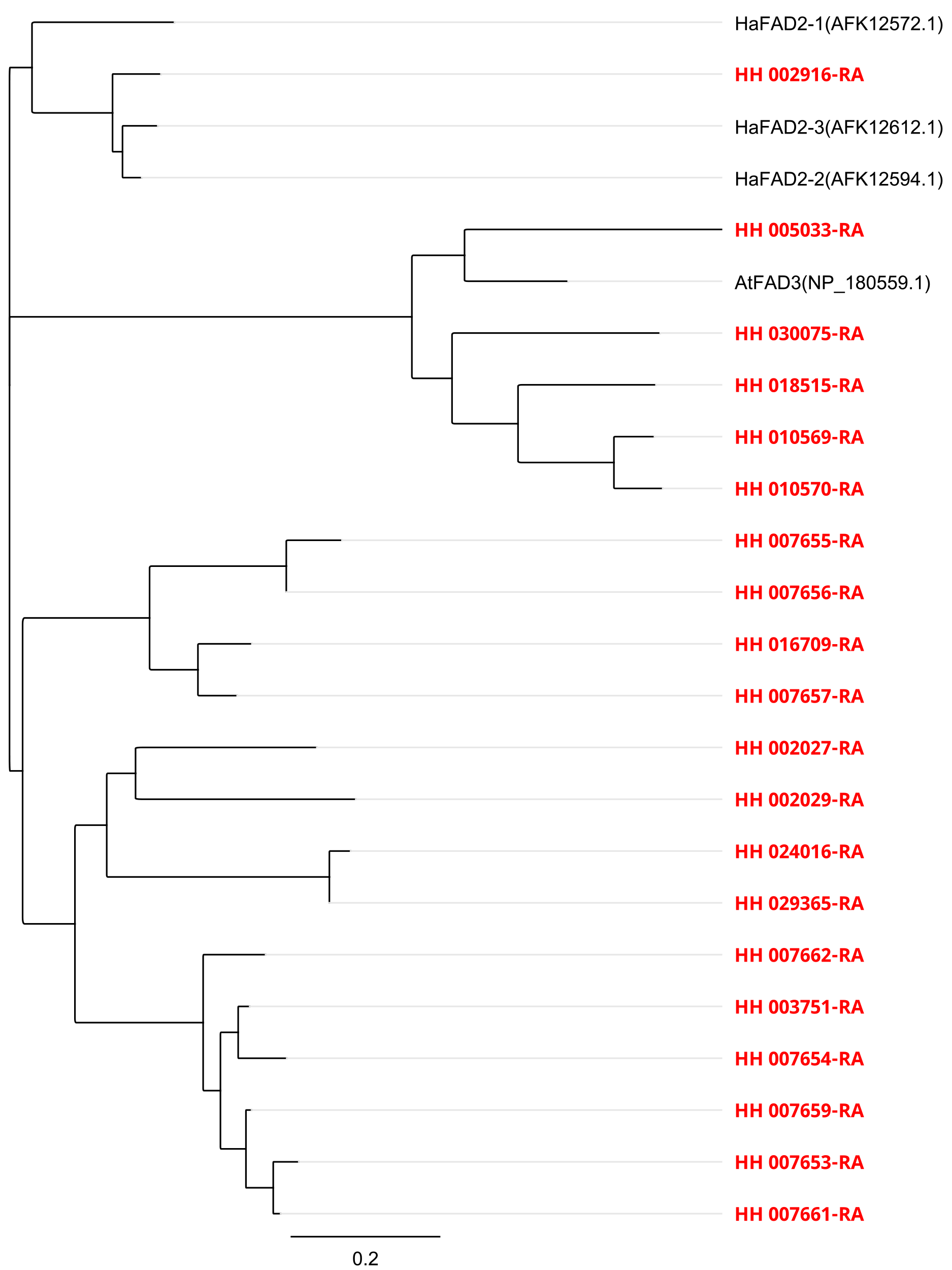


Figure S8 The speculative pathway of HSYA


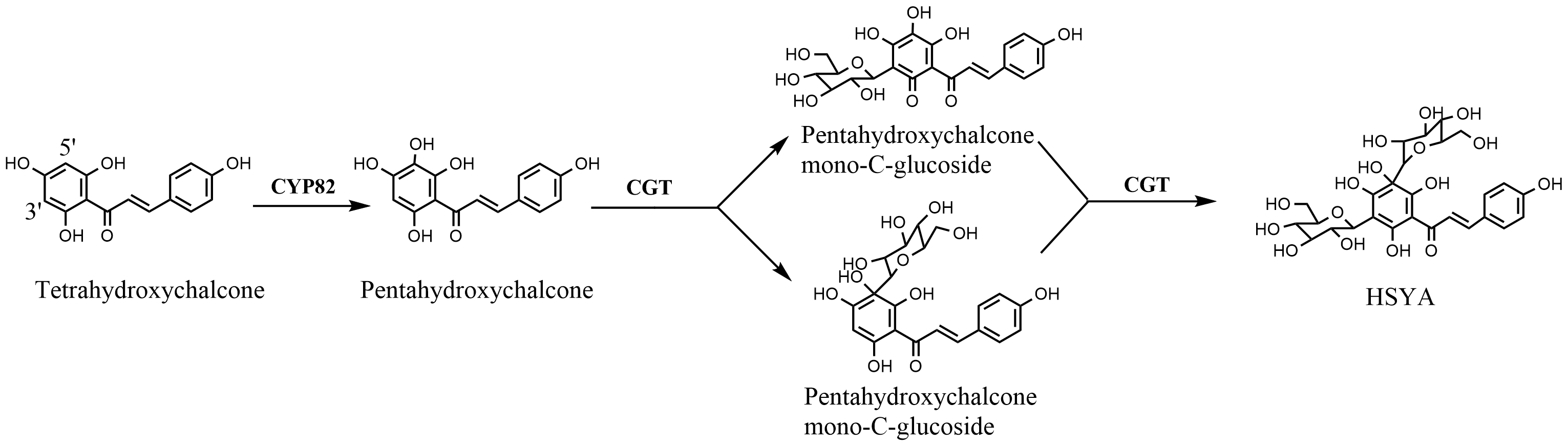


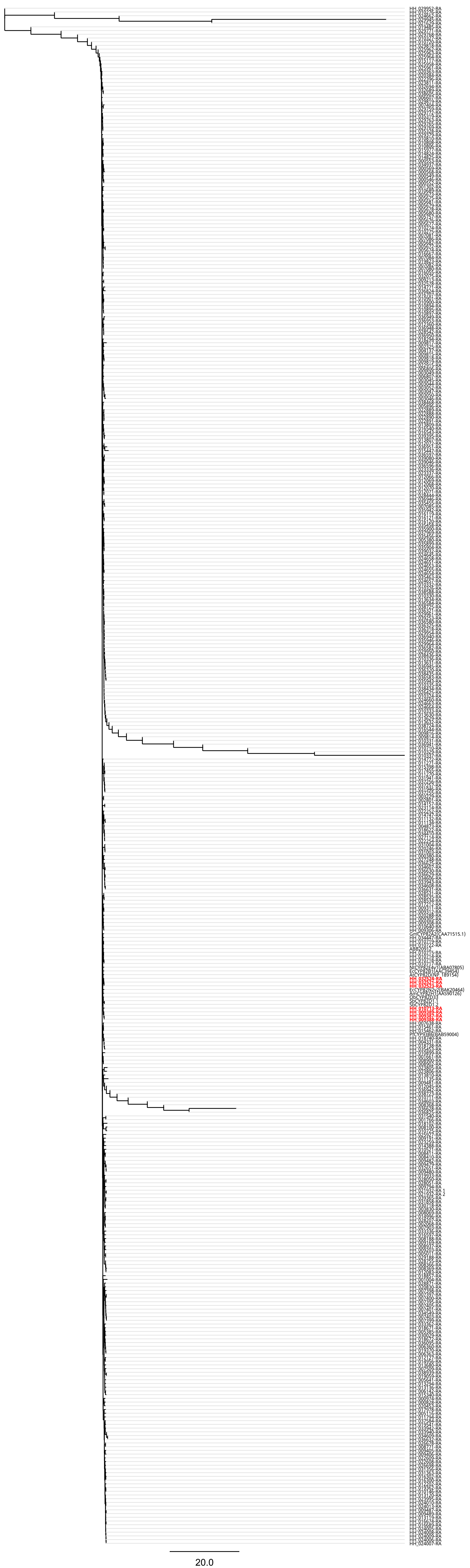


Figure S9 CYPs in safflower blasted with the reported CYP82. All CYPs in safflower were extracted with the pfam domainthe pfam domain PF00067

Figure S10 Phylogenetic analysis of CYPs in safflower with the homologies gene of CYP82D from *G. max*, *H. annuus* and *Arabidopsis*


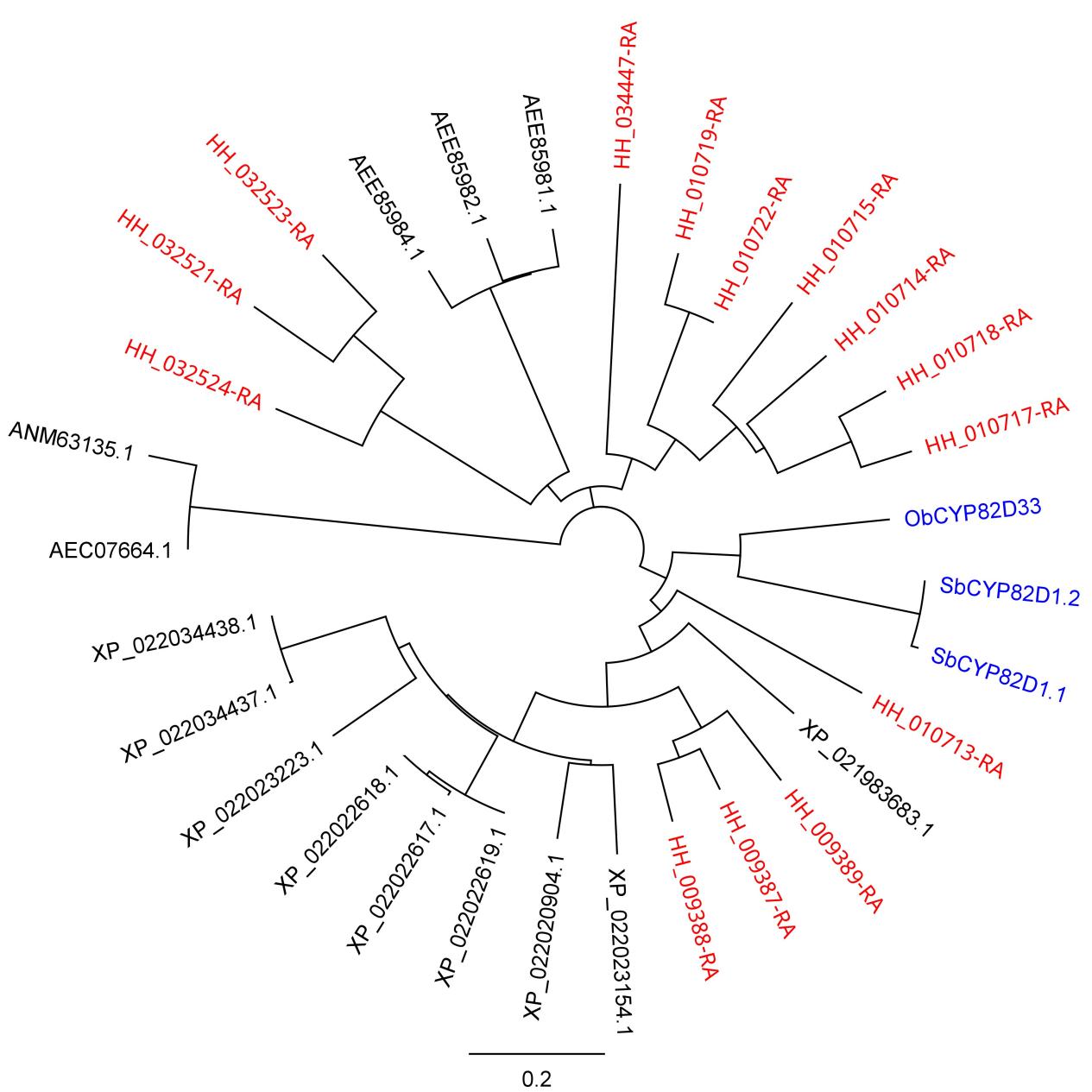


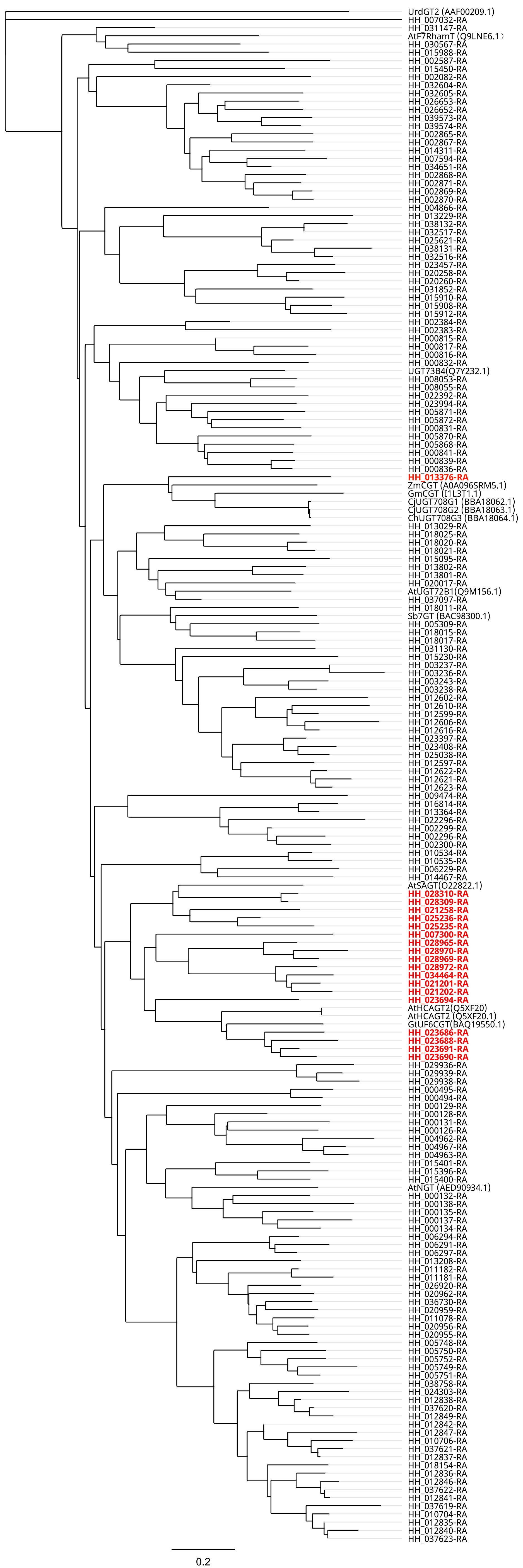


Figure S11 UGTs in safflower blasted with the reported UGTs. All UGTs in safflower were extracted with the pfam domain PF00201

Figure S12 Phylogenetic analysis of UGTs in safflower with the homologies gene of UGTs from *G. max*, *H. annuus* and *Arabidopsis*


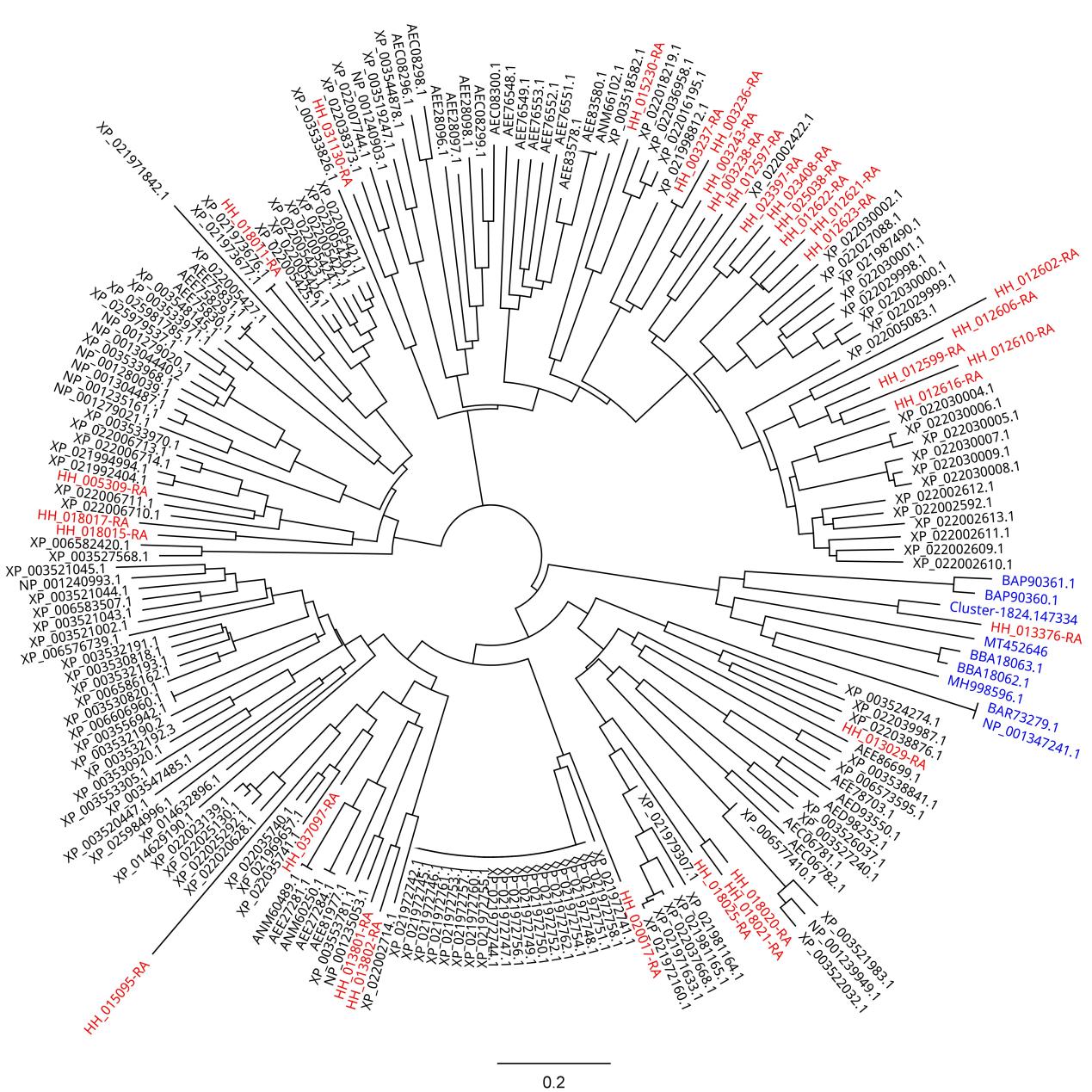


Figure S13 PCA analysis by the use of the high-quality SNP loci


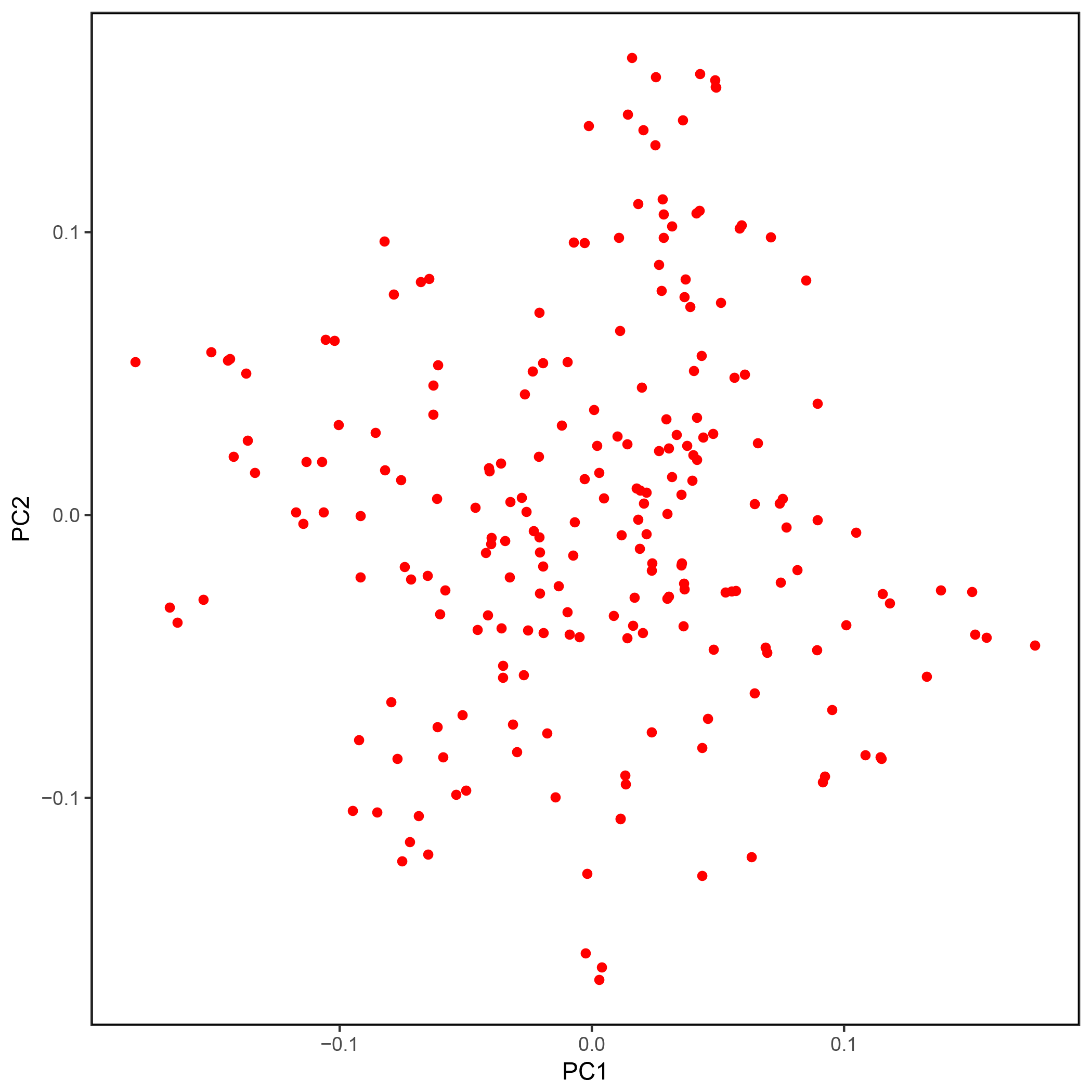


Figure S14 The population structure analysis with different K values in ADMIXTURE software


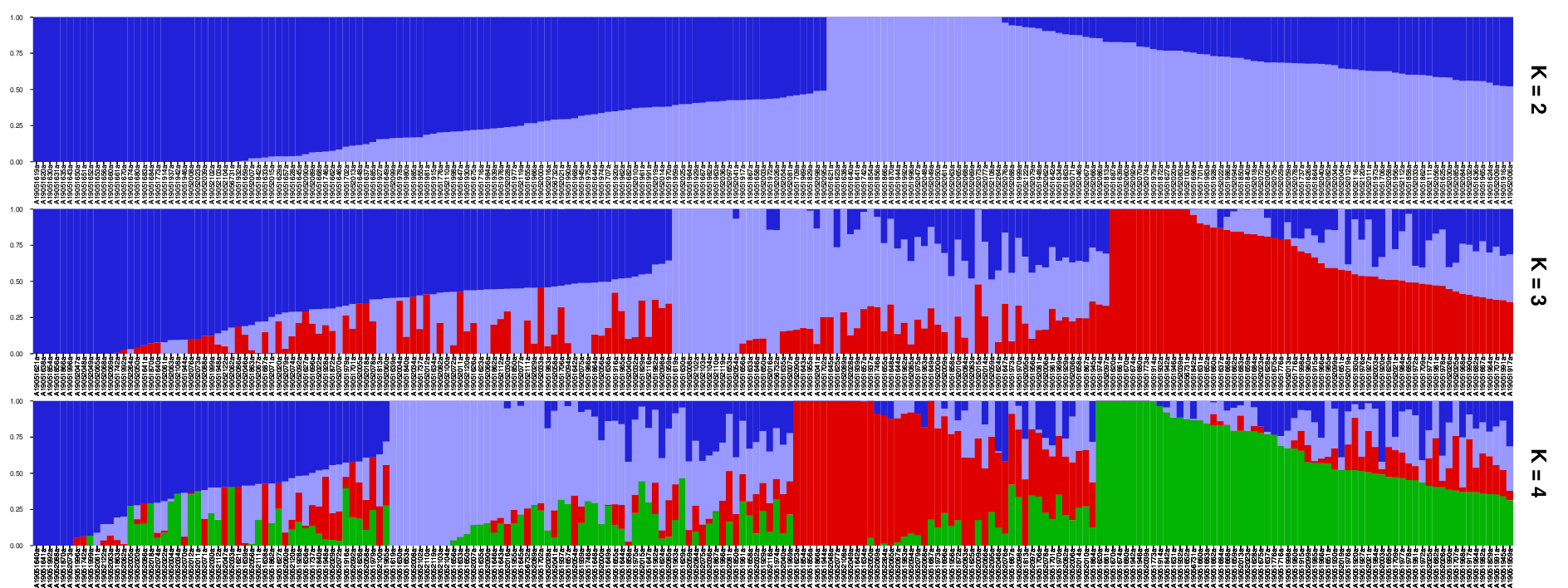


Figure S15 GWAS analysis for the the traits of BH, BN, BS, FBN, PH and SD. A is for the trait of BH (branch height); B is for the trait of BN (ball number); C is for the trait of BS (bract spine); D is for the trait of FBN (first branch number); E is for the trait of PH(Plant height); F is for the trait of SD (stem diameter)


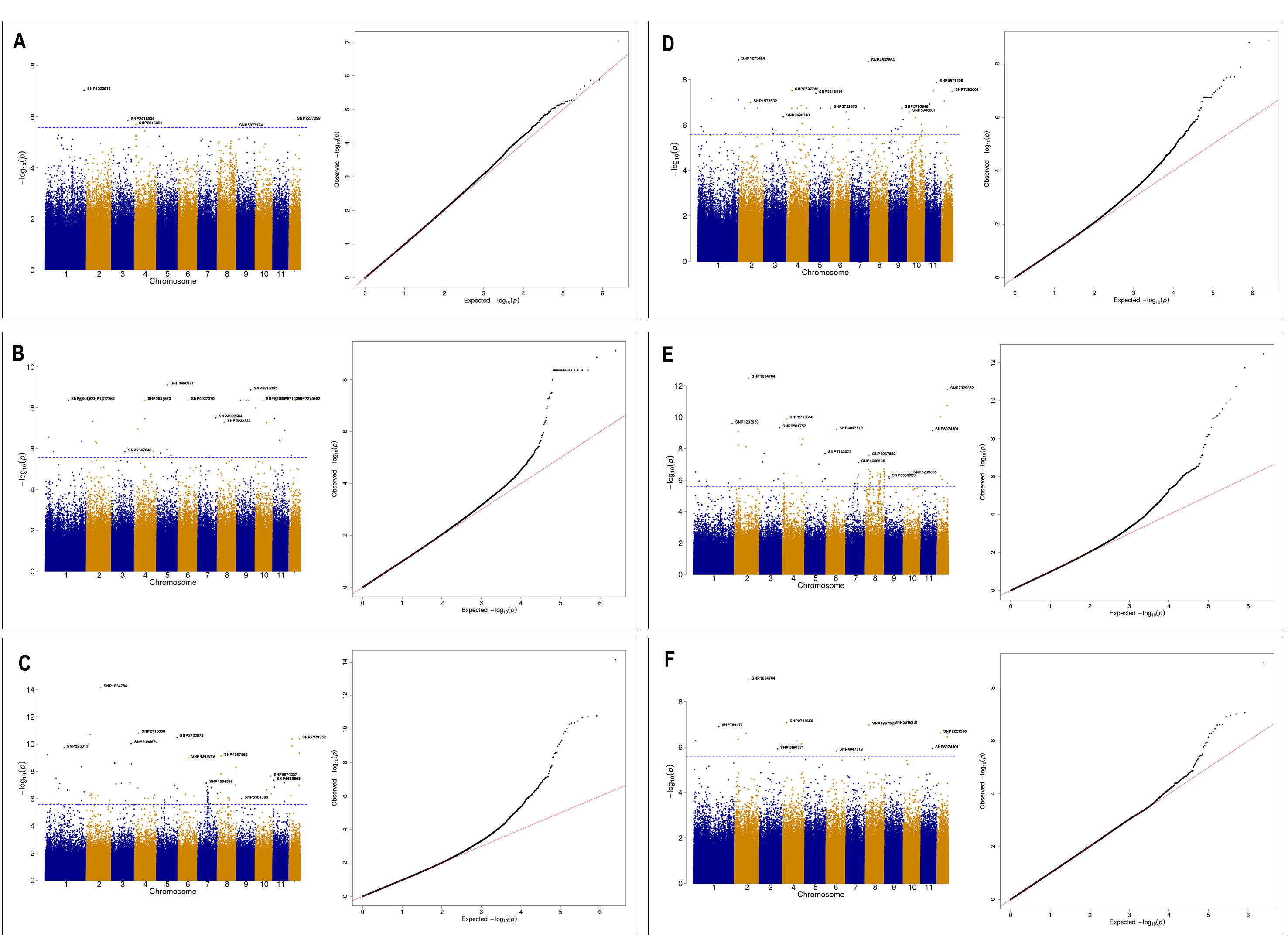


Figure S16 MS2 of products formed by the reaction of CtCGT1 with apigenin and naringenin. A The MS2 of the product formed by the reaction of CtCGT1 with apigenin, which is identical to isovitexin. B The MS2 of the product formed by the reaction of CtCGT1 with naringenin, which is identical to naringenin 6-C glycosides.

A

**
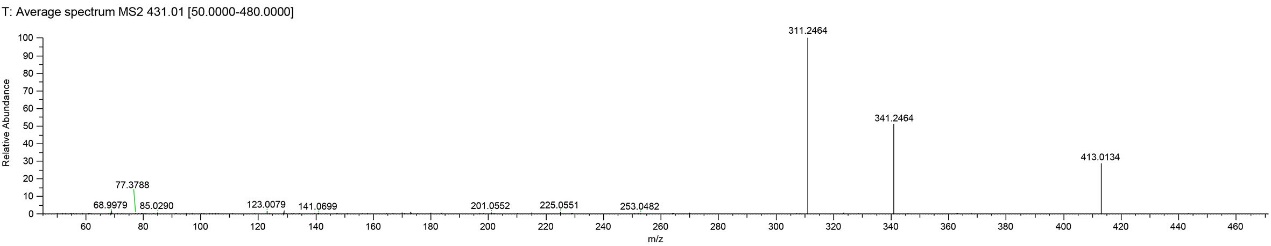

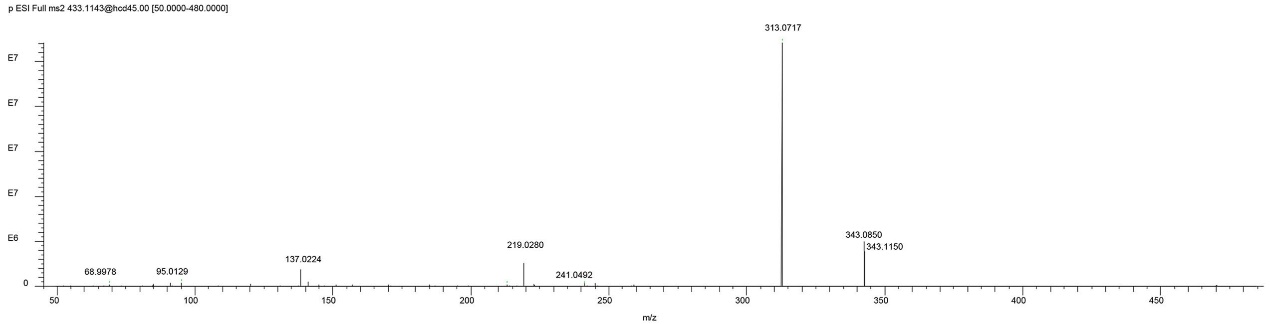
**

B
